# Comprehensive mutations analyses of *FTO* (fat mass and obesity-associated gene) and their effects on FTO’s substrate binding implicated in obesity

**DOI:** 10.3389/fnut.2022.852944

**Published:** 2022-07-18

**Authors:** Rakesh Kumar, Somorjit Singh Ningombam, Rahul Kumar, Harsh Goel, Ajay Gogia, Sachin Khurana, S. V. S. Deo, Sandeep Mathur, Pranay Tanwar

**Affiliations:** ^1^Laboratory Oncology Unit, Dr.B.R.A. Institute Rotary Cancer Hospital, All India Institute of Medical Sciences, New Delhi, India; ^2^Department of Medical Oncology, Dr.B.R.A. Institute Rotary Cancer Hospital, All India Institute of Medical Sciences, New Delhi, India; ^3^Department of Surgical Oncology, Dr.B.R.A. Institute Rotary Cancer Hospital, All India Institute of Medical Sciences, New Delhi, India; ^4^Department of Pathology, All India Institute of Medical Sciences, New Delhi, India

**Keywords:** obesity, FTO, missense mutation, MD simulation, networking, docking

## Abstract

An excessive amount of fat deposition in the body leads to obesity which is a complex disease and poses a generic threat to human health. It increases the risk of various other diseases like diabetes, cardiovascular disease, and multiple types of cancer. Genomic studies have shown that the expression of the fat mass obesity (*FTO*) gene was highly altered and identified as one of the key biomarkers for obesity. This study has been undertaken to investigate the mutational profile of the *FTO* gene and elucidates its effect on the protein structure and function. Harmful effects of various missense mutations were predicted using different independent tools and it was observed that all mutations were highly pathogenic. Molecular dynamics (MD) simulations were performed to study the structure and function of FTO protein upon different mutations and it was found that mutations decreased the structure stability and affected protein conformation. Furthermore, a protein residue network analysis suggested that the mutations affected the overall residues bonding and topology. Finally, molecular docking coupled with MD simulation suggested that mutations affected FTO substrate binding by changing the protein-ligand affinity. Hence, the results of this finding would help in an in-depth understanding of the molecular biology of the *FTO* gene and its variants and lead to the development of effective therapeutics against associated diseases and disorders.

## Introduction

The prevalence of obesity has been increasing worldwide, which poses a serious threat to human health (1). Obesity promotes various diseases such as diabetes, cardiovascular disease, and multiple types of cancer including breast, endometrial, blood, and ovarian cancer (2, 3). Several environmental factors such as lack of physical activity and higher consumption of energy-rich diets are primary obesogenic factors in the pathogenesis of obesity (4). Obesity is a complex phenotype, which is not a typical mendelian transmission and is a consequence of several susceptible genes with low or medium effects (5). It is a well-known fact that genes regulating energy homeostasis and thermogenesis, adipogenesis, leptin-insulin signaling transduction, and hormonal signaling peptides play an important role in the progression of obesity (6). There are different candidate genes which are reported to be associated with obesity. Among them, a fat mass and obesity-associated (*FTO*) gene was reported to be highly dysregulated and largely contributed to obesity (7, 8).

The Genome-wide association studies (GWAS) have shown that various single nucleotide polymorphisms (SNPs) of the *FTO* gene were strongly associated with increased body mass index (BMI) (9, 10). In addition to that, SNPs of the *FTO* gene were also found to be associated with various types of human cancer, such as breast, colon, gastric, pancreatic, and prostate cancer (11–15). The *FTO* gene is located in chromosome 16q12.2, with a total length of 410.50 kb including 9 exons and 8 introns (16). The dysregulation of *FTO* was involved in the impairment of different biological processes, such as proliferation, migration, invasion, cell cycle, and stem cell self-renewal through modulating various signaling pathways (17–19). *FTO* was identified as the first RNA demethylase that catalyzes oxidative demethylation of two different RNA nucleosides such as N6-methyladenosine (6mA) and 3-methyl uracil (m3U) *in vitro* and *in vivo*, respectively (20, 21).

Single nucleotide polymorphisms are the most common type of genetic alterations that occur both in coding and non-coding regions of DNA sequences, out of which, the mutations that occur in the coding part of genes directly affect the encoded protein (22). However, it also depends upon the nature of mutations like synonymous or non-synonymous where the former results in no alteration or later changed in the structure and hence the function of the protein (23). In this study, we have attempted to investigate the mutational profile of the *FTO* gene and to monitor the effect of non-synonymous (missense) mutations on the structure, dynamics, conformation, and substrate binding of the FTO protein. Different mutations were obtained through online databases and their pathogenicity was predicted through multiple online tools. Hence after, the impact of highly deleterious mutations on protein structure and functions was elucidated through molecular dynamics simulation, protein residues network, and molecular docking approaches.

## Materials and methods

### Dataset

The reported *FTO* SNPs and clinically important mutations were collected from dbSNP and ClinVar databases, respectively (24). The information about the functionally important mutations at the protein level was obtained from the UniProt database (25). Different mutations obtained from all databases were further verified by literature resources (8, 9, 26, 27). Tertiary structures of FTO protein are available at the protein data bank (PDB) with PDB codes: 3LFM and 5ZMD (21, 28). Since available structures at PDB are incomplete and have missing amino acids up to 35, therefore, we adopted homology and *de novo* modeling approaches to construct the full-length model using I-TASSER server (29).

### Pathogenicity and stability predictions of nsSNPs

Sequence-based pathogenicity prediction of various mutations was performed by the PredictSNP server which facilitates eight prediction tools: MAPP, PhD-SNP, PolyPhen-1, PolyPhen-2, SIFT, SNAP, PANTHER, and PredictSNP (30). It predicts the consequences of mutations on protein function based on different parameters inferred from evolutionary, physicochemical, or structural characteristics as previously described (22). Stabilities of different missense mutations were predicted through the Mutpred2, I-Mutant2.0, MUpro, mCSM, and iStable tools. MutPred2 predicts the pathogenicity of amino acid substitution and describes the molecular alterations likely altering the phenotype. It assesses the structural, functional, and phenotypic consequences of sequence variants. MutPred2 score > 0.5 indicated harmful mutation (31). I-Mutant2.0 is based on the support vector machine (SVM) that predicts protein stability upon substituting specific amino acid. I-Mutant2.0 predicts the direction of the free energy change (ΔΔG) values (kcal/mol). ΔΔG < 0 indicates the variant decreased the protein stability while a ΔΔG > 0 indicates the variant elevated protein stability (32). MUpro is also based on both the SVM and neural networks that predicts the effects of single-site mutation on protein stability. Free energy changes (ΔΔG) < 0 and ΔΔG > 0 indicate the variant with decreased and increased the protein stabilities, respectively (33). mCSM is a mutation cutoff scanning matrix tool which uses graph-based signatures to predict the impact of mutations on protein stability (34). Further, iStable server was also used to assess the stability of different mutants, and functionally important residues were predicted through an algorithm based on sequence conservation (35, 36).

### Preparation of mutant models and molecular dynamics simulation protocol

The tertiary model of FTO obtained from I-TASSER was considered a wildtype (WT) and different mutant (MT) models were constructed through the PyMOL graphical software (The PyMOL Molecular Graphics System, Version 1.3 Schrodinger, LLC). Both WT and MT models were initially energy minimized by GROMACS (Groningen Machine for Chemical Simulation) suite (37) and later the structure quality of all models was examined through the PROCHECK, ProSA, ERRAT, and QMEAN servers (38–41).

Molecular dynamics (MD) simulations were performed using the GROMACS program (37). The Amber03 force field was applied to generate the topologies of WT and all MTs (42). All the protein systems (WT and MTs) were solvated in a triclinic cubic box using the TIP3P (transferable intermolecular potential 3P) water model, under the defined buffer system of 0.12 nm distance between the edge of the protein to the surface of the box. Each system maintained electroneutrality with the addition of sodium (Na^+^) and chloride (Cl^–^) ions. Once the systems were neutralized, the energy minimizations were followed through the steepest descent (SD) method. Two common ensembles, NVT and NPT were employed using positional restraints for 100 and 500ps under a constant temperature of 300K and pressure of 1bar, respectively. The linear constraint solver (LINCS) algorithm was used to constrain all the bonds. The temperature and isotropic pressure of the system were maintained using modified Berendsen thermostat (v-scale) and Parrinello-Rahman methods, respectively. Further, the well-equilibrated complex molecules were subjected to a production run of 100ns time for each complex system in duplicate, by applying 2fs time steps. The analyses of trajectories were done through various utilities of GROMACS such as gmx energy, gmx rms, gmx rmsf, and gmx gyrate.

### Essential dynamics

To obtain a precise view of the prominent motions in a trajectory generated during MD simulation, we employed essential dynamics (ED) also known as principal component analysis (PCA). ED is a computational approach to understanding the correlated motions of a given protein that relate to its biological functions (43). The covariance matrix was constructed after eliminating the rotational as well as translational motions and diagonalized to generate a set of eigenvectors with respective eigenvalues. ED analysis was performed by gmx covar and gmx anaeig utilities of the GROMACS suite which were used to calculate and analyze the eigenvectors and their corresponding eigenvalues. Eigenvectors also known as principal components (PCs) help us to gain insight into the direction of concerted motions of an atom while its corresponding eigenvalues represent the magnitude of displacement. ED analyses were confined to backbone atoms to avoid statistical noise and motions owing to biological events were measured by assessing the cosine content of respective PCs (44).

### Protein network analysis

The functions of protein rely on its residues and interactions among them or with their surrounding environment. Each tertiary structure of protein exhibits its territory of network. To elucidate how non-synonymous mutations influenced the interatomic synergy, we studied residue interaction network (RIN) using the RINalyzer tool (45). Initially, MD optimized models were used as inputs to establish networks based on their physicochemical properties and their centralities. Network centrality was measured through weighted graph theory in which nodes represent residues and weight stand for the number of hydrogen bonds among the nodes. The obtained networks were analyzed and visualized in Cytoscape3.4 (46). We evaluated betweenness centrality (C_*B*_) for both WT and MTs which helped us to identify the essential residues imparted during signaling.

### Protein-ligand docking

Molecular docking is a key approach to studying the interaction between protein and small molecules such as ligand and the dynamics of that molecule within the binding pocket of the protein. Here, we used ensemble docking with 5 docking tools such as AutoDock Vina (47), QuickVina2 (48), SMINA (49), LeDock (50), and GNINA (51) and ligand binding affinity was measured in kilocalorie per mole. For all docking tools, except LeDock, the receptor and ligands were prepared in AutoDock tools as described previously (52, 53). Briefly, the receptor (WT and all MTs) and ligand were prepared by adding the polar hydrogen and Gasteiger charge, respectively. Dimension and coordinates for the ligand binding site were taken from the experimentally known binding site available at the PDB (5ZMD) structure (21). To cover the binding pocket, we created the grid box with the dimensions of 28 × 28 × 28 and centered at 80.533, 76.699, and 74.23 of x, y, and z coordinates, respectively. Receptor for LeDock was prepared in LePro module of LeDock tool and dimensions of grid box were set with the same size as mentioned above. Average docking scores and complexes with best binding poses for WT and all MTs were further analyzed. 3D and 2D plots of protein and protein-ligand systems were rendered in PyMOL (The PyMOL Molecular Graphics System, Version 1.3 Schrodinger, LLC), Schrodinger Maestro 12.8 (Maestro, Schrodinger, LLC, New York, NY, United States, 2021), and LigPlot + tools, respectively (54).

## Results

### Characterization of missense mutations of FTO gene

**FIGURE 1:**
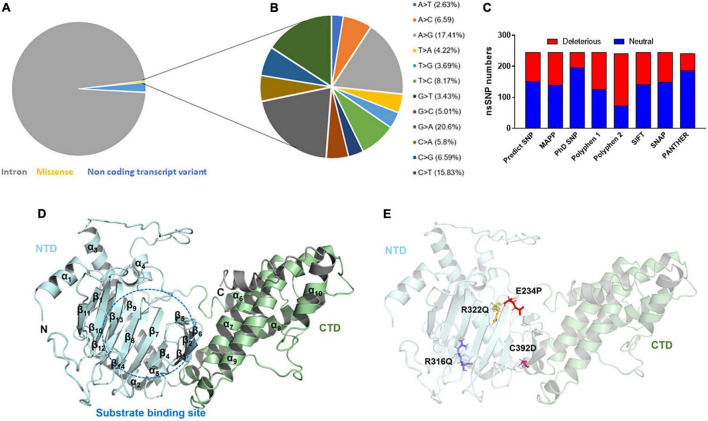
Mutational profile and tertiary structure of FTO protein. **(A)** Types of mutations, **(B)** total transition and transversion of non-synonymous missense mutations, **(C)** number of deleterious and neutral non-synonymous SNP, **(D)** tertiary structure of full length FTO protein showed different domains and **(E)** tertiary structure of protein showed various mutations. Protein structures were depicted in cartoon mode and substrate binding site was highlighted in blue dotted circle. Helices and sheets were labeled in α and β symbols. Different MTs such as E234P, R316Q, R322Q, and C392D were shown in red, blue, mustard and magenta color sticks, respectively. NTD, N-terminal domain; CTD, C-terminal domain.

The variants or mutations of *FTO* gene investigated in this study were retrieved from the dbSNP database. The database exhibited approximately 94,000 SNPs out of which about 91,312, 2,133, 383, 169, 2, and 1 SNPs were intronic, non-coding transcript variants, non-synonymous (missense), synonymous, inframe deletion, and initiator codon variants, respectively (Figure 1A). For further analysis, we have carried missense mutations as these mutations led to changes in amino acid residues that directly affect the structure and function of a protein. Further, the percentages of transition and transversion within the missense mutations of the *FTO* gene were inspected and it was found that the G > A (20.6%) transition showed the highest prevalence followed by A > G (17.41%), C > T (15.83%), and T > C (8.17%) (Figure 1B) while transversion such as A > C (6.59%) and C > G (6.59%) contributed the highest percentage followed by C > A (5.8%), G > C (5.01%), T > A (4.22%), T > G (3.69%), G > T (3.43%), and A > T (2.63%) (Figure 1B).

After removing the redundancy, about 245 missense variants were further used for the PredictSNP server to examine the deleterious nature of mutations (Figure 1C and Supplementary Table 1). Out of 245 variants, 7 variants (R96M, Y108A, F114D, E234P, R316Q, R322Q, and C392D) were finally selected based on higher frequencies of deleterious predicted by all 8 SNP tools (PredictSNP, MAPP, PhD-SNP, PolyPhen-1, PolyPhen-2, SIFT, SNAP, and PANTHER) (Supplementary Table 2). Additionally, the pathogenic strength and stability of 7 variants were further inspected through multiple independent tools such as I-Mutant, MuPro, mCSM, and iStable. Mutpred2 showed out of 7 variants, 6 were scored higher than 0.5 suggesting the pathogenic nature of these mutants. The highest score was observed in the F114D (0.926) variant followed by Y108A (0.926) and R96M (0.921), respectively (Table 1). ΔΔG prediction by I-mutant showed that all the selected nsSNPs or variants showed decreased stability (ΔΔG < 0) (Table 2). However, the lowest stabilities were found in the F114D and Y198A variants with ΔΔG values −3.13 and −2.44, respectively. MU-Pro analysis suggested that all the selected 7 variants were found to decrease protein stabilities, where Y108A (ΔΔG: −2.20) and F114D (ΔΔG: −1.78) showed the highest protein disabilities. According to the MU-Pro and mCSM analyses, all the selected variants were found to decrease stabilities except for the E234P variant which has a stabilizing property as predicted by mCSM. In mCSM, destabilizing was observed in all variants. Among them, Y108A (ΔΔG: −2.696) and F114D (ΔΔG: −2.694) variants showed a highly destabilizing nature (Table 2). The iStable server showed that all 4 MTs, namely E234P, R316Q, R322Q, and C392D exhibited decreased stabilities. The 3 variants (R316Q, R322Q, and C392D) unanimously showed ΔΔG < 1 kcal/mol calculated by three tools (I-Mutant, MuPro, and iStable), which would predict to disturb the structure and function of the protein. Functional impacts of R96M, Y108A, and F114D MTs were previously done through a structural-based experiment while the functional impact of 4 MTs such as E234P, R316Q, R322Q, and C392D remains to be explored (21). Therefore, in this study, we have elucidated the structural and functional impacts of 4 MTs namely E234P, R316Q, R322Q, and C392D on the FTO protein. Further, sequence conservation analysis showed that the Glutamate, Arginine, and Cysteine residues positioned at 234, 316, 322, and 392, respectively, exhibited ≥ 0.8 scores and thus indicated that the above residues are functionally conserved (Supplementary Table 3).

**TABLE 1:** Functional annotation of FTO variants.

Variants	MutPred2 score	Molecular consequences	Probability	*P*-value
**R96M**	0.921	Altered Ordered interface	0.37	3.0e-03
		Loss of Relative solvent accessibility	0.33	4.0e-03
		Altered DNA binding	0.32	1.7e-03
		Gain of Loop	0.29	7.0e-03
		Loss of Allosteric site at R96	0.26	0.01
		Altered Transmembrane protein	0.23	1.9e-03
		Altered Metal binding	0.20	0.04
**Y108A**	0.926	Altered Ordered interface	0.35	2.3e-03
		Altered Transmembrane protein	0.26	1.2e-03
		Loss of Acetylation at K107	0.21	0.04
		Loss of Allosteric site at Y106	0.20	0.04
		Altered Stability	0.20	0.01
		Altered DNA binding	0.16	0.16
		Gain of Catalytic site at R112	0.10	0.04
**F114D**	0.943	Loss of Strand	0.27	0.03
		Gain of Relative solvent accessibility	0.26	0.03
		Altered Transmembrane protein	0.25	1.3e-03
		Altered Ordered interface	0.24	0.04
		Altered DNA binding	0.22	0.01
		Gain of Allosteric site at R112	0.20	0.04
		Altered Stability	0.13	0.03
**E234P**	0.598	Altered Metal binding	0.52	3.6e-03
		Altered Ordered interface	0.24	0.05
		Gain of Allosteric site at W230	0.20	0.04
**R316Q**	0.237	–	–	–
**R322Q**	0.504	–	–	–
**C392D**	0.727	Altered Ordered interface	0.25	0.02
		Altered Disordered interface	0.19	0.04
		Altered Transmembrane protein	0.13	0.02

**TABLE 2:** Stability analysis of FTO variants.

Variants	I Mutant		MuPro		mCSM		iSTABLE	
	ΔΔG	Stability	ΔΔG	Stability	ΔΔG	Stability	ΔΔG	Stability
**E234P**	−0.08	Decrease	−1.06	Decrease	0.00	Stabilizing	−0.24	Decrease
**R316Q**	−1.36	Decrease	−0.80	Decrease	−1.40	Destabilizing	−0.47	Decrease
**R322Q**	−1.32	Decrease	−0.59	Decrease	−1.36	Destabilizing	−0.53	Decrease
**C392D**	−0.78	Decrease	−1.10	Decrease	−1.53	Destabilizing	−0.46	Decrease

### 3D structure of FTO consists of two distinct domains

FTO is ∼559 amino acid long protein (Uniprot: Q9C0B1) and its crystal structure is available at PDB which was resolved at ∼2.5Å resolution. However, the above reported structure has missing residues at flanking ends of N- (∼31) and C-terminals (∼62). Thus, we computationally modeled the full-length tertiary structure of FTO using the I-TASSER server service. The predicted tertiary model has −0.59 and 0.64 C- TM-scores, respectively, which indicate that the model is reliable and has a better global topology. Further reliability of the generated model was verified by superposing the template and model (Supplementary Figure 1). We omitted ∼31 and 38 residues long extreme N- and C-terminals as these residues formed highly disordered structures and also these regions do not affect the conformation and catalytic activity of the protein. The tertiary structure of FTO comprised 9 α-helices and 14 β-sheets. Double-stranded distorted β sheets reside solely in NTD along with 4 α helices (α1–α4) while CTD consists of 5 unique helices (α5–α9). NTD controls core catalytic activity with the involvement of CTD and encompasses β5–β14 (Figure 1D). CTD is primarily made up of α-helices in which α7, α8, and α10 formed a three-helix bundle. One end of the helix interacts with NTD thus involved in the stabilization of NTD conformation. Mutations in the CTD domain greatly affected the conformation and catalytic activity of the protein (28). Out of 4 selected mutations, 3 mutations (E234P, R316Q, and R322Q) were located at NTD while 1 mutation (C392D) was located at CTD (Figure 1E). The tertiary model obtained from I-TASSER was assigned as wildtype (WT) and structures of all 4 mutants (MTs) were prepared in the PyMOL tool using WT as templates. All WT and MT models were further subjected to quality assessment by evaluation of their 3D geometry and stereochemical properties. The model quality evaluation results suggested that WT and all MTs had a negligible number of amino acids at the disallowed region of Ramachandran plots and had high structure quality as indicated by > 85% of ERRAT scores for WT and all MT models (Supplementary Table 4).

### System and structure stability analyses

**FIGURE 2:**
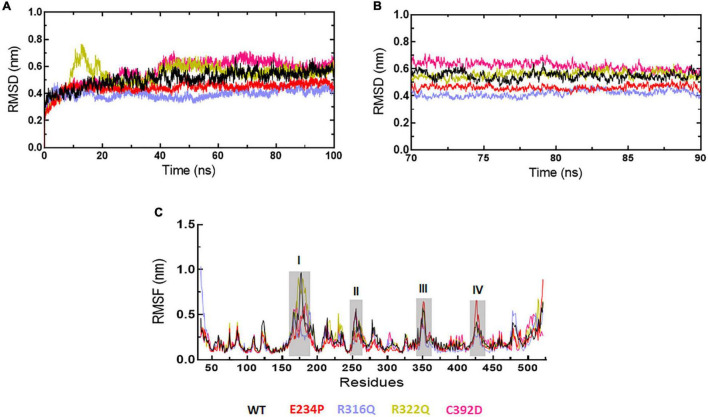
MD simulation profiles of WT and MT proteins. **(A)** Root mean square deviation (RMSD) profiles of WT and different MTs measured in nanometers at function of time, **(B)** RMSD profiles of WT and different MTs from equilibrated trajectories of 70–90 ns time periods (20 ns total) measured in nanometers at function of time, and **(C)** Root mean square fluctuations (RMSF) of different MTs along with WT measured in nanometers. Highly fluctuated protein residues were shown in shaded areas and labeled in numbers. WT, E234P, R316Q, R322Q, and C392D were labeled in black, red, blue, mustard, and magenta color, respectively.

The sequence level prediction of protein stability was examined through various tools as discussed above. The protein stabilities at the structure level of WT and all MTs were assessed through MD simulations which were performed in duplicate for 100 ns each (Supplementary Figure 2). During MD simulations various parameters such as RMSD, RMSF, and Rg were measured concerning equilibrium structures. RMSDs of WT and MTs were thoroughly stabilized within a time scale of 100 ns (Figure 2A). WT and all MTs showed steady behaviors of RMSDs after ∼60ns time. Hence, well-equilibrated trajectories were utilized for further analysis in all cases (Figure 2B). The average RMSD values of WT, E234P, R316Q, R322Q and C392D were approximately 0.50, 0.44, 0.39, 0.54, and 0.56 nm, respectively (Table 3). E234P and R316Q MTs showed higher RMSDs than WT with 13 and 22 nm of percent differences, respectively, while R322Q and C392D MTs showed lower RMSDs than WT with percent differences of −8 and −12 nm, respectively. The Above data showed that RMSDs of all MTs displayed stable behaviors with more or fewer RMSD values as compared to WT indicating that E234P, R316Q, R322Q, and C392D MTs largely affected the structure of the FTO protein. The effect of different mutations on the protein compactness and size was monitored by measuring the Rg at the function of time. WT and all MTs showed a similar pattern of Rg with average values being ∼2.64, 2.62, 2.62, 2.63, and 2.63 nm for WT, E234P, R316Q, R322Q, and C392D, respectively (Supplementary Figure 3). All MTs displayed similar Rg values with WT indicating that the compactness of the FTO protein remains unaffected upon these mutations.

**TABLE 3:** Mean RMSD and Rg of WT and MTs and the percentage difference between them.

	RMSD (nm)	Percent difference (WT-MT)	Rg (nm)	Percent difference (WT-MT)
WT	0.50	0	2.64	0
E234P	0.44	13	2.62	1
R316Q	0.39	22	2.62	1
R322Q	0.54	−8	2.63	0.5
C392D	0.56	−12	2.63	0.5

### Root mean square fluctuations analyses

Next, we wanted to check the stabilities and flexibilities of WT and MT proteins at residues and secondary structure levels. To accomplish this, we measured root mean square fluctuation (RMSF) and secondary structures (α-helix, β-sheet, bridge, turn, coil, and bend) of WT and MT proteins for individual residue and domain throughout the simulation period. The fluctuation intensities varied among different regions of WT and all MTs proteins (Figure 2C). The regions occupied around positions I (160-190), II (250-260), III (345-355), and (IV) (420-430 residues) showed > 0.5nm of RMSFs in WT and all MTs. WT showed maximum fluctuation in region I (∼1 nm) and E234P showed maximum fluctuation at region IV (∼0.6 nm) (Figure 2C). The R322Q MT showed maximum fluctuation in region I (∼0.9nm). The R316Q, R322Q, and C392D MTs showed fewer fluctuations in regions II and IV thus implying that these MTs decreased the flexibilities of the given segments of the FTO protein. Further to investigate the effect of different mutations on the secondary structures of the FTO protein, a dictionary of secondary structure of protein (DSSP) was performed during MD simulation. It was found that the coil contents in all MTs were increased as compared to WT (Supplementary Table 5). The increased coil content led to decreased protein flexibility which was also observed during the RMSF analyses.

### Structure–function relationship

**FIGURE 3:**
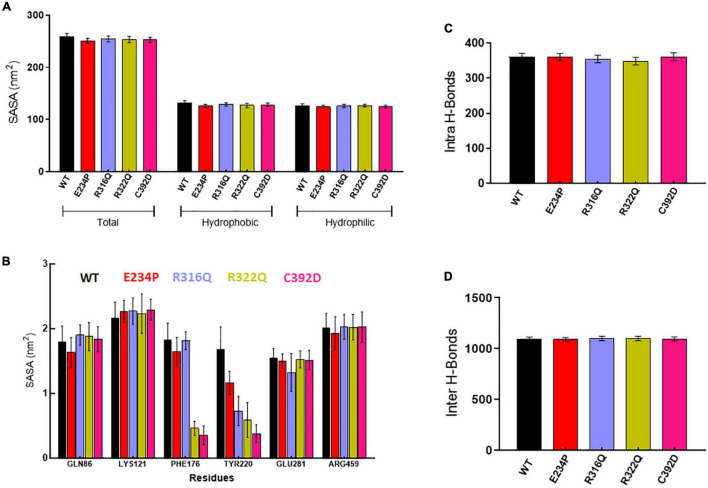
Structural properties analyses of WT and MT proteins. **(A)** Total, hydrophobic and hydrophilic solvent accessible surface area plot, **(B)** solvent accessible surface area of protein residues, **(C)** total number of intra or protein-protein hydrogen bond and **(D)** total number of inter or protein-water hydrogen bond. WT, E234P, R316Q, R322Q, and C392D were labeled in black, red, blue, mustard, and magenta color, respectively.

Further, we wanted to study the structural and functional properties of various MTs along with WT by calculating the solvent-accessible surface area (SASA), protein–protein (intra), and protein–water (inter) hydrogen bonding. Overall, SASA was further divided into hydrophobic and hydrophilic SASA which showed steady behaviors throughout the simulation period in WT as well as in all MTs (Supplementary Figure 4). The total mean values of SASA for WT, E234P, R316Q, R322Q, and C392D were approximately 258.7, 250.6, 254.4, 253.4, and 252.7 nm^2^, respectively (Figure 3A) All MTs displayed lesser values of SASA as compared to WT. This suggested that all MTs have a low surface area of exposure for the interaction with other molecules. WT, E234P, R316Q, R322Q, and C392D exhibited average hydrophilic SASA values of about 126.4, 124.6, 125.7, 126.6, and 124.6 nm^2^, while hydrophobic SASA of all MTs remained similar to WT. Furthermore, SASA per residues basis was calculated, and it was found that GLN86, LYS121, PHE176, TYR220, GLU281, and ARG459 exhibited higher SASA values than the rest of the amino acid residues in WT and all MTs (Figure 3B).

Wild Type and MTs showed consistent behaviors of intra- and inter-H-bonds formation (Supplementary Figure 5). The average intra-H-bonds of WT, E234P, and C392D displayed a similar number of H-bonds (360) while R316Q (354) and R322Q (348) MTs showed a lesser number of intra-H-bonds as compared to WT (Figure 3C). Lesser number of inter-H-bonds were found in E234P (1090) MTs as compared to WT (1092), while R316Q (1101) and R322Q (1100) MTs exhibited higher mean values of inter-H-bonds (Figure 3D). The above results showed that R316Q and R322Q MTs exhibited lesser intra-H-bonds indicating the above MTs are more flexible and less stable. Moreover, these MTs exhibited higher inter-H-bonds indicating that the above MTs had higher interacting capacities as compared to WT.

### Mutations in NTD affected the conformation of CTD

**FIGURE 4:**
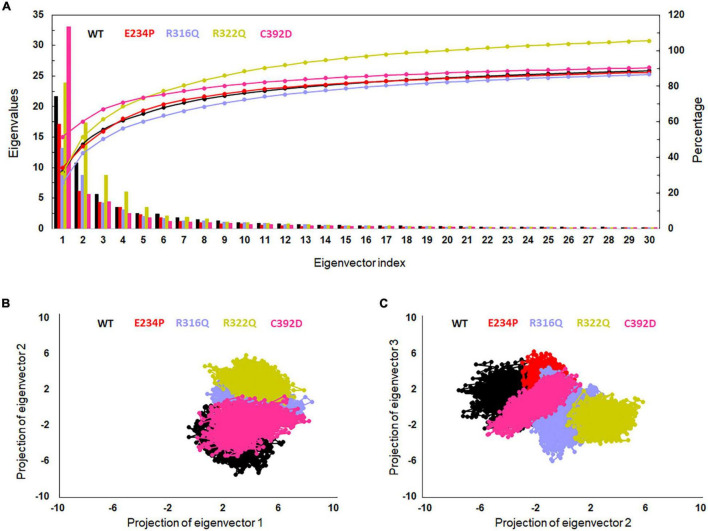
Essential dynamics profile of WT and MT proteins. **(A)** Plot of first 30 eigenvectors with corresponding eigenvalues. **(B)** Projection of eigenvector1 versus eigenvector2 and **(C)** projection of eigenvector2 versus eigenvector3. WT, E234P, R316Q, R322Q, and C392D were labeled in black, red, blue, mustard, and magenta color, respectively.

**FIGURE 5:**
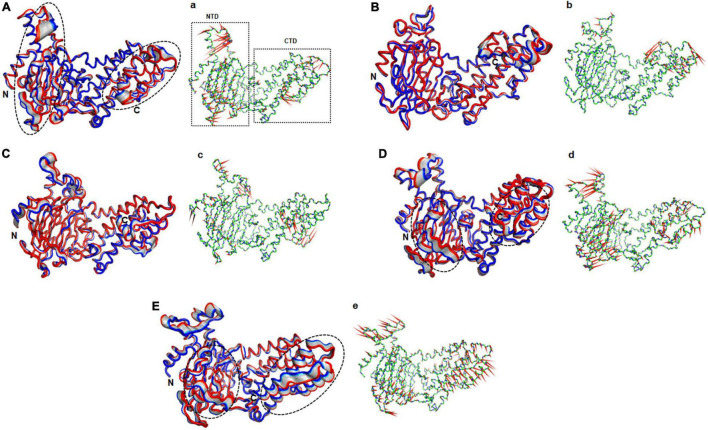
Essential motions analyses of WT and MT proteins. **(A)** WT, **(B)** E234P, **(C)** R316Q, **(D)** R322Q, and **(E)** C392D. Motions in both NTD and CTD were highlighted in black dotted circles. Red to blue regions indicated minimum to maximum motions, respectively. Panels (a–e) showed corresponding porcupine structures. Length and direction of cones in the porcupine structures represented magnitude and direction of the motions. NTD, N-terminal domain; CTD, C-terminal domain.

**FIGURE 6:**
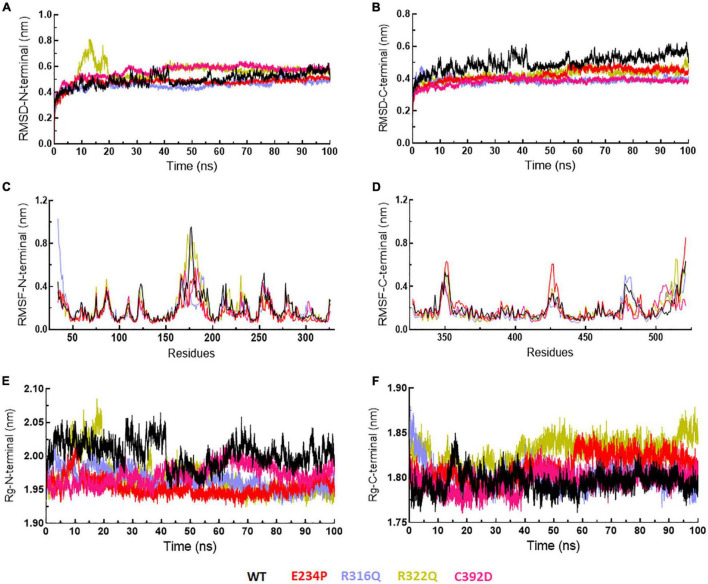
MD simulation profiles of N- and C-terminal domains of WT and MT proteins. **(A,B)** RMSD profiles of N- and C-terminal domains, **(C,D)** RMSF profiles of N- and C-terminal domains, and **(E,F)** radius of gyration plots of N- and C-terminal domains. WT, E234P, R316Q, R322Q, and C392D were labeled in black, red, blue, mustard, and magenta color, respectively.

The function of protein depends upon its motion which is elucidated by conformational sampling in phase space from MD simulated trajectories. To study the appropriate motions of the protein resulting from atomic trajectories, we performed essential dynamics and reduce the dimension of data using principal component analysis (PCA). R322Q MT exhibited relatively higher trace values of covariance matrix while E234P, R316Q, and C392 MTs consisted of lower matrix values as compared to WT suggesting that the R322Q MT accompanied higher motions as compared to WT (Supplementary Table 6). During PCA, we examined the first 30 eigenvectors with corresponding eigenvalues which showed cumulative percentage of more than 85% in WT and all MTs (Figure 4A and Supplementary Table 6). In conjunction with the first 30 eigenvectors, we observed that the first 3 eigenvectors or PCs shared more than 50% of the cumulative percentage, which plays a significant role in the motions of protein (Supplementary Table 6). Moreover, to further confirm the movement of protein is not due to random diffusion, we investigated cosine value, in which a lower value (<0.2) was observed in the first 2 PCs (Supplementary Table 7). Further, to understand the protein conformation, we projected eigenvector 1 versus eigenvector 2 and eigenvector 2 versus eigenvector 3 in phase spaces (Figures 4B,C). In all 3 projections (PC1, PC2, and PC3), E234P occupied a smaller subspace in comparison to WT suggesting that it restricted the motions of the FTO protein (Figures 4B,C). Furthermore, to understand the motions at domain or 3D structural levels, we utilized 30 frames of the first eigenvector by sequentially superimposing the corresponding frames (Figure 5). We found that the motions were found at both N- and C-terminal domains. MTs such as E234P and R316Q (Figures 5B,C) have fewer motions in both domains, while R322Q and C392D MTs (Figures 5D,E) showed more motions as compared to WT (Figure 5A). Interestingly, mutations in the N-terminal domain (NTD) affected the motions of both NTD and the C-terminal domain (CTD). Motions were limited to loop and turn regions of N- and C-terminal domains (Figures 5a–e). The results of PCA are quite interesting as it could differentiate the properties of different MTs instead of having similar patterns of RMSD (Figures 6A,B and Supplementary Table 8), RMSF (Figures 6C,D), and Rg (Figures 6E,F) of NTD and CTD.

### Betweenness centrality and residues interaction network analyses

**FIGURE 7:**
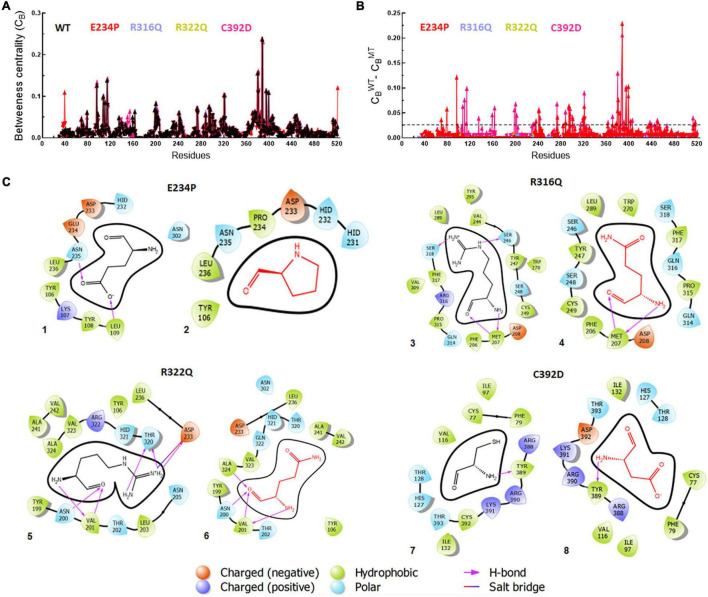
Betweenness centrality (C_*B)*_ and residues interactions analyses of WT and MT proteins. **(A)** Individual betweenness centralities of MTs along with WT, **(B)** difference in betweenness centralities of WT and MTs and **(C)** 2D interaction maps of native and mutant residues. E234P (1 and 2), R316Q (3 and 4), R322Q (5 and 6) and C392D (7 and 8). Straight dotted line in panel **(B)** indicated cut-off C_*B*_ value which should be less than 0.02. Native and mutant residues were labeled in black and red, color, respectively, while different charge residues and bonding were depicted in different color as shown above in the figure.

Betweenness centrality (C_*B*_) analysis was performed to understand the key residues involved in the function of the protein. During this analysis, the residues in the protein were represented as nodes while the connections among these residues were denoted as edges. Here, we calculated 3 main centralities: betweenness centrality (C_*B*_), closeness (C_*C*_), and degree (C_*D*_) for WT and all MTs out of which C_*B*_ has the significance of calculating the importance of each residue in the signaling of proteins upon different mutations. We mapped the residues having C_*B*_ ≥ 0.05 in WT and all MTs and found a similar number of residues involved in signaling (Figure 7A). Similarly, we have calculated the difference between the C_*B*_ values of WT and MTs and the residues satisfying C_*B*_ ≥ 0.02 (Figure 7B and Supplementary Table 9). These residues were mainly confined to the catalytic core and NTD and CTD of the FTO protein (Supplementary Figure 6). In this analysis, 28 and 40 residues play a significant role in the signaling of E234P and C392D MTs, respectively (Figure 7B and Supplementary Table 9), while no residues satisfying the conditions ≥ 0.02 existed in R316Q and R322Q MTs. This suggested that substitution of Arginine at 316 and 322 with Glutamine largely influenced the residue signaling of the FTO protein. Moreover, all individual MTs affected the dynamics and stabilities of different MTs (Supplementary Figure 7)

Furthermore, the residues-residues interactions network of WT and all MTs were plotted to investigate different types of bonding. To accomplish this task, a Cytoscape plugin with RINalyzer was employed in which nodes were represented as residue and different types of bonding such as van der Waals (VDW), hydrogen bonds (H-bond), pi-pi, and ionic interactions were represented as edges (Supplementary Figure 8). Further, a 2D interaction map of mutated residues was analyzed to examine the different types of interactions with surrounding residues and compared with native (Figure 7C). The van der Waals and hydrogen bonds were mainly affected in the E234P MT. The VDW interactions were reduced in all MTs except R316Q while R316Q displayed a similar number of VDW interactions with respect to WT (Figure 7C and Supplementary Figure 8). However, the H-bonds were decreased in R316Q and R322Q MTs as compared to WT while the H-bonds in C392D MT remained unaffected. The Pi-pi stack bonding was also reduced in R322Q MT. The residues network analysis results indicated that all non-covalent interactions were affected and impaired in almost all MTs.

### Mutations affected substrate binding

**FIGURE 8:**
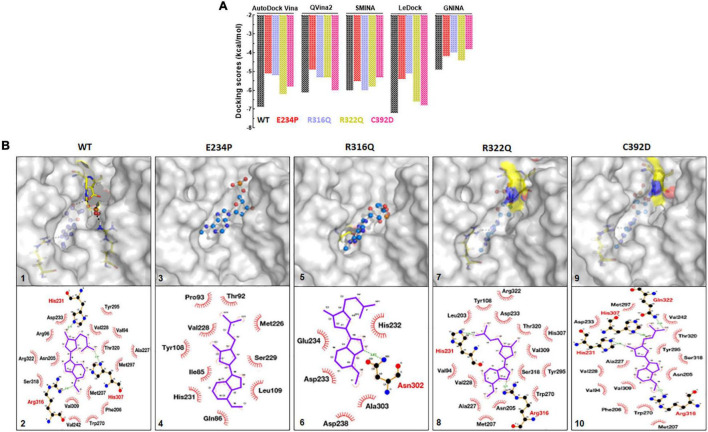
Protein-ligand docking and interactions analyses of WT and MTs. **(A)** Docking scores from AutoDock Vina, QVina2, SMINA, LeDock and GNINA docking tools. **(B)** 3D and 2D interaction maps of (1) and (2) WT-6mA, (3) and (4) E234P-6mA, (5) and (6) R316Q-6mA, (7) and (8) R322Q-6mA and (9) and (10) C392D-6mA. WT, E234P, R316Q, R322Q and C392D were labeled in black, red, blue, mustard, and magenta color, respectively. In 3D interaction map, protein and ligand were shown in surface and ball and stick mode, respectively. In 2D interaction maps ligand shown in blue color and hydrophilic and hydrophobic interacting residues were labeled in red and black color.

FTO and its substrate binding were monitored by calculating the protein-ligand binding affinity using the docking approach which was measured in kcal/mol. N6-Methyl-deoxy-adenosine-5′-monophosphate (6mA) is one of the major substrates of FTO and we used this as a ligand for measuring the binding affinity upon different mutations. A combination of 5 docking tools was employed for WT- and MT-6mA complexes and it was found that all docking tools showed a similar pattern of docking scores even when all docking tools have different scoring functions (Figure 8A). The average docking scores of WT, E234P, R316Q, R322Q, and C392D were −6.2, −5.02, −5.1, −5.6, and −5.5 kcal/mol, respectively (Supplementary Table 10). The docking results indicated that all MTs exhibited high docking scores compared to WT suggesting that all MTs showed low binding affinities to the substrate than WT. Further to understand the residues participating in both hydrophobic and hydrophilic interactions during ligand binding, we analyzed the 3D and 2D plots of high-scored protein-ligand complexes (Figure 8B). The results suggested that 18 bonds (hydrophobic:15; hydrophilic:3) existed between the WT- and C392D-6mA complexes (Figure 8B). R322Q-6mA formed the maximum number of bonds (hydrophobic: 17; hydrophilic: 3) (Figure 8B) while E234P-6mA formed the minimum number of bonds (hydrophobic: 9; hydrophilic: 0) (Figure 8B). Further, different MTs led to a change in the electrostatic and hydrophobic nature of the core and binding regions of the protein compared with the WT-substrate complex (Supplementary Figure 9). The protein-ligand interaction studies indicated that binding affinities of almost all MT-ligand complexes were decreased which suggested that binding of the FTO substrate was largely affected by the given point mutations.

**FIGURE 9:**
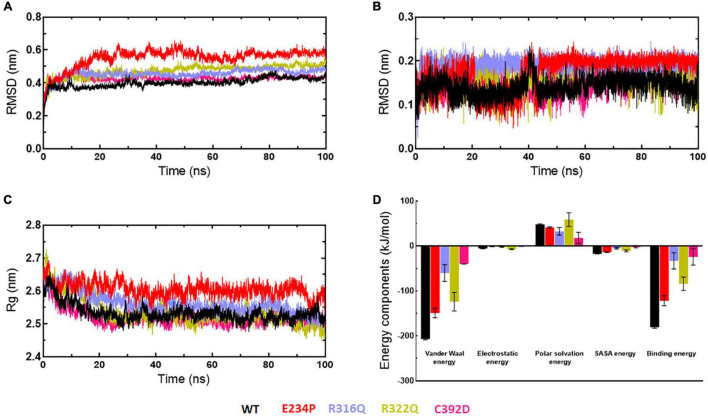
MD simulation profiles of WT and MT-6mA complexes. **(A)** RMSD profiles of WT and different MT proteins, **(B)** RMSD profiles of 6mA substrate in complexed with WT and different MTs, **(C)** radius of gyration plots of WT- and MT-6mA complexes, and **(D)** interaction energy plot of WT- and MT-6mA. WT, E234P, R316Q, R322Q, and C392D were labeled in black, red, blue, mustard, and magenta color, respectively. Different interaction energies were measured in kilocalorie per mole.

Stabilities and rescoring of binding energies of docking complexes (WT and MTs) were assessed through 100ns MD simulation (Figure 9). Binding energies of all docking complexes were calculated by g_mmpbsa script using the last 20ns-stabled MD simulated trajectories (55). Initially, the stabilities of WT- and MT-6mA complexes were assessed by monitoring the protein and drug RMSD behaviors followed by inspecting the Rg and RMSF of the protein in complexed form. RMSD of WT, R316Q, R322Q, and C392D showed steady behavior and stabilized after 40ns time while RMSD of E234P was stabilized after 70 ns simulation time (Figure 9A). RMSD of all WT and MT proteins in the complexed form displayed consistent and equilibrated behaviors. The stability of 6mA substrate in protein complexes was examined by measuring the ligand RMSD which showed steady behaviors after 45 ns simulation time in all cases (Figure 9B). Further, the compactness of protein in complexed form was monitored through the inspection of Rg behaviors and demonstrated that Rg of WT and all MT proteins displayed consistent and steady behaviors (Figure 9C). RMSF of protein in complexed form showed similar patterns as observed in the apo form (Supplementary Figure 10). The stability analyses of WT- and MT-6mA complexes suggested that all complexes were well stabilized throughout the simulation period. Binding energies of WT- and MT-6mA complexes were studied by calculating all energy components such as van der Waals (VDW), electrostatic, polar solvation, and solvent accessible surface area (SASA) energies. Binding energies of WT-, E234P-, R316Q-, R322Q-, and C392D-6mA were −180.911 ± 2.1, −121.83 ± 11, −32.92 ± 18.2, −83.78 ± 2.114.7, and −23.92 ± 18.5 kcal/mol, respectively (Figure 9D). The lowest binding free energy of the WT-6mA complex as compared to all MT-6mA complexes demonstrated that all MT-6mA complexes have low binding affinities than WT which also agreed with the docking results and VDW energy was the major contributor.

## Discussion

The current genomic era has enabled the researcher to elucidate the precise structure and functions of disease-associated mutations both at the gene and protein levels (56). Various diseases such as multiple cancer, obesity, and cardiovascular disease are arising due to point mutations (57–59). The *FTO* gene which is highly dysregulated and known to be involved in various types of cancer and obesity has been recently identified (60). The FTO protein plays a role in adipogenesis and cancer through the 6mA-dependent demethylase activity which affects various mRNA processing steps. It is involved in obesity development by influencing the 6mA level of eating-related hormones or certain adipogenesis-related chemicals. The overexpression of the *FTO* gene increased the energy intake by affecting the methylation of ghrelin mRNA (61). FTO controls the splicing of the exon of the adipogenic regulatory factor RUNXITI (RUNX1 translocation partner 1) and is known to be involved in adipogenesis through 6mA-YTHDF2 (YTH N6-Methyladenosine RNA Binding Protein 2)-dependent pathway *via* regulating cell cycle proteins (62, 63). Besides obesity, *FTO* has an important role in tumorigenesis and pathogenicity of different types of cancer. It promotes leukemic cell transformation and leukemogenesis through inhibiting ATRA (all-trans-retinoic acid)-induced AML (acute myeloid leukemia) cell differentiation and induced malignant characteristics in breast cancer cells by targeting BNIP3 (BCL2 Interacting Protein 3) (64, 65). Knockdown of *FTO* increases the level of 6mA which promotes cell proliferation and invasion by activating PI3K (Phosphatidylinositol-3-kinase) signaling in gastric cancer and increases lung squamous cell growth and invasiveness by inhibiting the cell apoptotic genes (66, 67). *FT*O promotes chemotherapy resistance by increasing the mRNA expression of β-catenin in cervical squamous cell carcinoma (68).

Various mutations that occur both at the exon and intron of *FTO* have been previously explored. Independent GWAS studies reported two different SNPs (rs9939609, rs9930506) in the first intron of the *FTO* gene significantly associated with BMI (69, 70). A further study conducted on the European population has identified another SNP (rs1558902) located in the same chromosome (60). Three GWAS studies in the East Asian populations, such as Korean, Chinese, and Japanese, have identified various mutations in the *FTO* gene (rs9939609, rs17817449, and rs12149832) as the most significant marker of BMI (69, 70). In addition to this, six SNPs of the *FTO* gene (rs6499640, rs1421085, rs8050136, rs3751812, rs9939609, and rs9930506) were mostly associated with obesity (71, 72). Besides the polymorphism of *FTO* found in obesity, it also contributed to many other diseases among various populations. A case-control study in the Chinese Han population found that different variants of *FTO* gene like the C variant of rs1421085, T variant of rs3751812, A variant of rs8050136, and rs9939609, were associated with metabolic dysfunction-association fatty liver disease (69). Enormous studies on the gene level have been accomplished to cover the intronic and exonic profiles of *FTO*, but none of the studies has explored the functional or protein product of gene alterations. Additionally, a large genomic study covering an entire pool of exonic variations has never been performed and the mutations reflecting the structure and functions of protein remain to be explored.

In this study, we have attempted to investigate the functional consequences of all non-synonymous mutations of the *FTO* gene occurring in the public database. Transition (Ti) and transversion (Tv) analyses from all non-synonymous missense mutations showed that the G > A and A > C were highly prevalent Ti and Tv, respectively. After that sequence and structure levels prediction of pathogenic mutations were analyzed through a pile of tools and it was observed that most of the mutations were pathogenic and deleterious. Around 245 missense mutations were analyzed out of which 7 mutations (R96M, Y108A, F114D, E234P, R316Q, R322Q, and C392D) were shown to be highly pathogenic. Previous experimental studies have suggested that these mutations led to various disease disorders. Structural, conformational, and functional consequences of the above mutations were further inspected through 3D structural modeling and molecular dynamics simulation approaches. The tertiary structure of FTO protein was constructed through the I-TASSER server. The I-TASSER has been proved to be the best protein structure prediction tool by CASP (Critical assessment of structure prediction) analysis (73). Recently, a neural network-based tool such as AlphaFold has been introduced for modeling the tertiary structures of protein (74). Structures from both servers (I-TASSER and AlphaFold) were compared with the PDB structure and found low RMSDs suggesting that the modeled structures were very similar to the experimental ones. The tertiary structure of the FTO protein is mainly comprised of two large N- and C-terminal domains with both consisting mixtures of helices and sheets. NTD exhibited the catalytic activity of the protein and CTD assisted in conformational movement during substrate binding. Thus, both domains have functional importance. The predicted model was having a better 3D geometry and good global topology. The structural and functional analyses of WT and MTs were further examined by calculating the RMSD, Rg, and RMSF using the MD simulation technique. MD simulation is commonly applied for refining the computational model, to assess the stability of WT and MT proteins, to study the conformational changes of the protein that are essential for function, and to calculate the binding energy of ligand (22, 75). RMSD of all WT and MTs were measured at the function of time in 100ns and found that the RMSDs were well stabilized. Further, Rg of WT and MTs were measured to study the effect of different MTs on the compactness and globularity of the FTO protein and it was observed that the various mutations had not affected the protein compactness. Structural fluctuation and stability at the global and local levels were inspected by measuring the RMSF and secondary structures of WT and MTs that formed. The results suggested that fluctuations were mainly restricted to loop or turn regions of the protein and coil content of all MTs was increased, thus, impairing the overall flexibility of the protein.

The structure and functional impacts of various MTs on the protein were elucidated by examining the SASA, intra- and inter-H-bonds. SASA helps to determine the conformation of protein through its accessibility in the surrounding environment which mediates its interaction with the solvent molecule. The SASA results suggested that the lower surface area available for interactions was accompanied by almost all the MTs as compared to WT. Hydrogen bond plays a crucial role in protein stability, protein folding, and molecular interactions (76). Intra-H-bonds (protein–protein) provide overall stability and shape of the protein, while inter-H bonds (protein–solvent) confer the capacity of formation of intermolecular interactions of proteins. The H-bond results suggested that MT exhibited lower intra-H-bond as compared to WT, thus, indicating that MTs had deceased stabilities. Stable conformation of proteins is crucial for their function and such conformations are better studied by essential dynamics technique. The conformational effect of different mutations on the FTO protein was analyzed by gmx covar and anaeig modules of GROMACS. During this analysis, we observed that major motions were accompanied by the first 30 eigenvectors in all WT and MTs and restricted mainly to the N- and C-terminal domains. The E234P and R316Q MTs showed rigid motions in both domains as compared to WT, while C392D MT showed higher motions. Mutations at NTD largely affected the motions of CTD which imply that both domains are essential for the stable conformation of the protein. Further, to gain insight into the protein architecture like residue-residue interactions, a residues network analysis was conducted. In this analysis, betweenness centrality (C_*B*_), which is a parameter to identify crucial residues involved in the protein structure and functions, was measured for WT and all MT proteins. The results suggested that substitution of ARG316 and ARG322 to GLN316 and GLN322 largely affected the residues network of the FTO protein. In addition to that, residue-residue bindings were also analyzed and it was found that H-bonding and all non-covalent interactions were largely affected and impaired in MTs as compared to WT.

Considering the contributing role of 6mA modification on gene expression, it has been enormously involved in various human diseases like psychiatric, metabolic syndrome, cardiovascular diseases, and various forms of cancer (77). It has been recognized as one of the post-transcriptional regulatory markers in different types of RNAs and plays an important role in RNA splicing, translation, stability, translocation, and high-level structure (78). The FTO protein interacts with 6mA through its catalytic core site which mainly occurs in the NTD (20). Next, we analyzed whether different missense mutations affected the binding of FTO and 6mA, therefore, we performed molecular docking of 6mA with both WT and MT proteins. The docking results from 5 docking tools suggested that MTs showed high docking scores as compared to WT, demonstrating the weaker binding affinities with 6mA, thus indicating the given mutations largely affected the FTO-6mA binding. Further, rescoring of binding energies of docking complexes through MD simulation demonstrated that MT-6mA complexes had low binding affinities than the WT-6mA complex which again indicated that the above mutations largely affected the FTO’s substrate binding.

The FTO protein consists of 2 domains located at the N-and C-terminals, and hence, known as N and C terminal domains (NTD and CTD) (21). The currently studied mutations were mainly located at NTD (E234P, R316Q, and R322Q) while one mutation such as C392D was located at CTD. Residues at NTD like E234, R316Q, and R322Q formed the part of the catalytic core in which substrates like 6mA binds with protein (28). Mutations in any residues of catalytic core distort the substrate-binding cavity which ultimately abolishes the function of the protein. In E234P MT, Glutamate (E), an acidic amino acid with a negatively charged side chain is replaced by Proline (P), which abolishes the side-chain H-bonding formed by the carboxylic group, thus forming an unstable structure that distorts the binding pocket. In R316Q and R322Q MTs, Arginine (R) is located in the double-strand β-helix of NTD, replaced with Glutamine (Q) having an amide group on their side chain. Arginine is a basic amino acid having a positive charge due to the presence of α-amino and imidazole groups on its side chain which mediates the interaction with the surrounding residues through multiple H-bonding and enhances the catalytic activity of the protein. Substitution of R with Q at position 316 reduces the H-bonding, while at position 322, all interactions such as H-bonding, van der Waals as well as ionic interactions are affected leading to distortion and instability of catalytic core, and hence, affecting the substrate binding which is evident from MD simulation and docking results. Mutations of R316Q and R322Q were reported to be associated with a malformation syndrome inherited in an autosomal recessive pattern (79). Finally, the C392D mutation located at the CTD interacts with NTD. Cysteine (C) forms disulfide bridges which stabilize the overall tertiary structure of the protein by inducing conformation changes both in the N- and C-terminal domains (28). Substitution of C at position 392 with Aspartate (D) abolishes the van der Waals interactions that were formed with Phenylalanine (F) 79 in NTD, and hence, destabilizes the overall structure. Moreover, this also disrupts the interaction between the N-and C-terminal domains which ultimately reduces the demethylase activity of the protein.

## Conclusion

The comprehensive mutational study of the *FTO* gene has shown that the missense mutations are highly pathogenic and largely affect the structure and conformation of the protein which ultimately affects the function of FTO. Furthermore, mutations also lead to amending the FTO substrate binding. To our knowledge, this is a prime study where the entire pool of *FTO* variants was conducted which will assist in understanding the mechanism of the associated diseases and disorders and would set a path for precise therapeutics.

## Data availability statement

The original contributions presented in this study are included in the article/Supplementary Material, further inquiries can be directed to the corresponding author.

## Author contributions

RakK, SN, and PT conceptualized and designed the study. SN and RakK performed SNP mining and annotation. RakK and RahK performed MD simulation and docking experiments. HG, AG, SK, SD, and SM helped in the literature study. RakK, SN, RahK, and HG analyzed the data and wrote the manuscript. RakK, SN, RahK, HG, and PT reviewed and approved the final version of the manuscript. All authors contributed to the article and approved the submitted version.

## Conflict of interest

The authors declare that the research was conducted in the absence of any commercial or financial relationships that could be construed as a potential conflict of interest.

## Publisher’s note

All claims expressed in this article are solely those of the authors and do not necessarily represent those of their affiliated organizations, or those of the publisher, the editors and the reviewers. Any product that may be evaluated in this article, or claim that may be made by its manufacturer, is not guaranteed or endorsed by the publisher.

## References

[1] Ng M Fleming T Robinson M Thomson B Graetz N Margono C Global, regional, and national prevalence of overweight and obesity in children and adults during 1980–2013: a systematic analysis for the Global Burden of Disease Study 2013. *Lancet*. (2014) 384:766–81. 10.1016/S0140-6736(14)60460-8 24880830 PMC4624264

[2] Calle EE Kaaks R. Overweight, obesity and cancer: epidemiological evidence and proposed mechanisms. *Nat Rev Cancer*. (2004) 4:579–91. 10.1038/nrc1408 15286738

[3] Goodwin PJ Stambolic V. Impact of the obesity epidemic on cancer. *Annu Rev Med*. (2015) 66:281–96. 10.1146/annurev-med-051613-012328 25423596

[4] Chauhdary Z Rehman K Akash MS. The composite alliance of FTO locus with obesity-related genetic variants. *Clin Exp Pharmacol Physiol*. (2021) 48:954–65. 10.1111/1440-1681.13498 33735452

[5] Yang W Kelly T He J. Genetic epidemiology of obesity. *Epidemiol Rev*. (2007) 29:49–61. 10.1093/epirev/mxm004 17566051

[6] Loktionov A. Common gene polymorphisms and nutrition: emerging links with pathogenesis of multifactorial chronic diseases. *J Nutr Biochem*. (2003) 14:426–51. 10.1016/s0955-2863(03)00032-9 12948874

[7] Rankinen T Zuberi A Chagnon YC Weisnagel SJ Argyropoulos G Walts B The human obesity gene map: the 2005 update. *Obesity*. (2006) 14:529–644. 10.1038/oby.2006.71 16741264

[8] Dina C Meyre D Gallina S Durand E Körner A Jacobson P Variation in FTO contributes to childhood obesity and severe adult obesity. *Nat Genet*. (2007) 39:724–6. 10.1038/ng2048 17496892

[9] Frayling TM Timpson NJ Weedon MN Zeggini E Freathy RM Lindgren CM A common variant in the FTO gene is associated with body mass index and predisposes to childhood and adult obesity. *Science*. (2007) 316:889–94. 10.1126/science.1141634 17434869 PMC2646098

[10] Scuteri A Sanna S Chen WM Uda M Albai G Strait J Genome-wide association scan shows genetic variants in the FTO gene are associated with obesity-related traits. *PLoS Genet*. (2007) 3:e115. 10.1371/journal.pgen.0030115 17658951 PMC1934391

[11] Da Cunha PA de Carlos Back LK Sereia AF Kubelka C Ribeiro MC Fernandes BL Interaction between obesity-related genes, FTO and MC4R, associated to an increase of breast cancer risk. *Mol Biol Rep*. (2013) 40:6657–64. 10.1007/s11033-013-2780-3 24091943

[12] Yamaji T Iwasaki M Sawada N Shimazu T Inoue M Tsugane S. Fat mass and obesity-associated gene polymorphisms, pre-diagnostic plasma adipokine levels and the risk of colorectal cancer: the Japan Public Health Center-based Prospective Study. *PLoS One*. (2020) 15:e0229005. 10.1371/journal.pone.0229005 32053666 PMC7017986

[13] Xu D Shao W Jiang Y Wang X Liu Y Liu X. FTO expression is associated with the occurrence of gastric cancer and prognosis. *Oncol Rep*. (2017) 38:2285–92. 10.3892/or.2017.5904 28849183

[14] Lin Y Ueda J Yagyu K Ishii H Ueno M Egawa N Association between variations in the fat mass and obesity-associated gene and pancreatic cancer risk: a case–control study in Japan. *BMC Cancer*. (2013) 8:337. 10.1186/1471-2407-13-337 23835106 PMC3716552

[15] Lewis SJ Murad A Chen L Davey Smith G Donovan J Palmer T Associations between an obesity related genetic variant (FTO rs9939609) and prostate cancer risk. *PLoS One*. (2010) 19:e13485. 10.1371/journal.pone.0013485 20976066 PMC2957440

[16] Lan N Lu Y Shuangshuang P Xi H Nie X Liu J FTO-a common genetic basis for obesity and cancer. *Front Genet*. (2020) 11:559138. 10.3389/fgene.2020.559138 33304380 PMC7701174

[17] Liu S Huang M Chen Z Chen J Chao Q Yin X FTO promotes cell proliferation and migration in esophageal squamous cell carcinoma through up-regulation of MMP13. *Exp Cell Res*. (2020) 389:111894. 10.1016/j.yexcr.2020.111894 32035950

[18] Zhang Z Zhou D Lai Y Liu Y Tao X Wang Q Estrogen induces endometrial cancer cell proliferation and invasion by regulating the fat mass and obesity-associated gene via PI3K/AKT and MAPK signaling pathways. *Cancer Lett*. (2012) 319:89–97. 10.1016/j.canlet.2011.12.033 22222214

[19] Huang H Wang Y Kandpal M Zhao G Cardenas H Ji Y FTO-dependent N6-methyladenosine modifications inhibit ovarian cancer stem cell self-renewal by blocking cAMP signaling. *Cancer Res*. (2020) 80:3200–14. 10.1158/0008-5472.CAN-19-4044 32606006 PMC7442742

[20] Wei J Liu F Lu Z Fei Q Ai Y He PC Differential m6A, m6Am, and m1A demethylation mediated by FTO in the cell nucleus and cytoplasm. *Mol Cell.* (2018) 71:973–985.e5. 10.1016/j.molcel.2018.08.011 30197295 PMC6151148

[21] Zhang X Wei LH Wang Y Xiao Y Liu J Zhang W Structural insights into FTO’s catalytic mechanism for the demethylation of multiple RNA substrates. *Proc Natl Acad Sci USA*. (2019) 116:2919–24. 10.1073/pnas.1820574116 30718435 PMC6386707

[22] Kumar R Kumar R Tanwar P Deo SV Mathur S Agarwal U Structural and conformational changes induced by missense variants in the zinc finger domains of GATA3 involved in breast cancer. *RSC Adv*. (2020) 10:39640–53. 10.1039/D0RA07786K 35515377 PMC9057444

[23] Yang Z Nielsen R. Synonymous and nonsynonymous rate variation in nuclear genes of mammals. *J Mol Evol*. (1998) 46:409–18. 10.1007/pl00006320 9541535

[24] Sherry ST Ward MH Kholodov M Baker J Phan L Smigielski EM dbSNP: the NCBI database of genetic variation. *Nucleic Acids Res*. (2001) 29:308–11. 10.1093/nar/29.1.308 11125122 PMC29783

[25] UniProt Consortium. UniProt: a hub for protein information. *Nucleic Acids Res.* (2015) 43:D204–12. 10.1093/nar/gku989 25348405 PMC4384041

[26] Rohena L Lawson M Guzman E Ganapathi M Cho MT Haverfield E FTO variant associated with malformation syndrome. *Am J Med Genet A.* (2016) 170A:1023–8. 10.1002/ajmg.a.37515 26697951

[27] Boissel S Reish O Proulx K Kawagoe-Takaki H Sedgwick B Yeo GS Loss-of-function mutation in the dioxygenase-encoding FTO gene causes severe growth retardation and multiple malformations. *Am J Hum Genet*. (2009) 85:106–11. 10.1016/j.ajhg.2009.06.002 19559399 PMC2706958

[28] Han Z Niu T Chang J Lei X Zhao M Wang Q Crystal structure of the FTO protein reveals basis for its substrate specificity. *Nature*. (2010) 464:1205–9. 10.1038/nature08921 20376003

[29] Zhang Y. I-TASSER server for protein 3D structure prediction. *BMC Bioinformatics*. (2008) 23:40. 10.1186/1471-2105-9-40 18215316 PMC2245901

[30] Bendl J Stourac J Salanda O Pavelka A Wieben ED Zendulka J PredictSNP: robust and accurate consensus classifier for prediction of disease-related mutations. *PLoS Comput Biol*. (2014) 10:e1003440. 10.1371/journal.pcbi.1003440 24453961 PMC3894168

[31] Pejaver V Urresti J Lugo-Martinez J Pagel KA Lin GN Nam HJ Inferring the molecular and phenotypic impact of amino acid variants with MutPred2. *Nat. Commun*. (2020) 11:5918. 10.1038/s41467-020-19669-x 33219223 PMC7680112

[32] Cpriotti E Fariselli P Casadio RI-. Mutant2. 0: predicting stability changes upon mutation from the protein sequence or structure. *Nucleic Acids Res*. (2005) 33:W306–10. 10.1093/nar/gki375 15980478 PMC1160136

[33] Cheng J Randall A Baldi P. Prediction of protein stability changes for single-site mutations using support vector machines. *Proteins*. (2006) 62:1125–32. 10.1002/prot.20810 16372356

[34] Pires DE Ascher DB Blundell TL. mCSM: predicting the effects of mutations in proteins using graph-based signatures. *Bioinformatics*. (2014) 30:335–42. 10.1093/bioinformatics/btt691 24281696 PMC3904523

[35] Chen CW Lin J Chu YW. iStable: off-the-shelf predictor integration for predicting protein stability changes. *BMC Bioinformatics.* (2013) 14(Suppl2):S5. 10.1186/1471-2105-14-S2-S5 23369171 PMC3549852

[36] Capra JA Singh M. Predicting functionally important residues from sequence conservation. *Bioinformatics.* (2007) 23:1875–82. 10.1093/bioinformatics/btm270 17519246

[37] Van Der Spoel D Lindahl E Hess B Groenhof G Mark AE Berendsen HJ. GROMACS: fast, flexible, and free. *J Comput Chem*. (2005) 26:1701–18. 10.1002/jcc.20291 16211538

[38] Laskowski RA Rullmann JA MacArthur MW Kaptein R Thornton JM. AQUA and PROCHECK-NMR: programs for checking the quality of protein structures solved by NMR. *J Biomol NMR*. (1996) 8:477–86. 10.1007/BF00228148 9008363

[39] Wiederstein M Sippl MJ. ProSA-web: interactive web service for the recognition of errors in three-dimensional structures of proteins. *Nucleic Acids Res*. (2007) 35:W407–10. 10.1093/nar/gkm290 17517781 PMC1933241

[40] Lüthy R Bowie JU Eisenberg D. Assessment of protein models with three-dimensional profiles. *Nature*. (1992) 356:83–5. 10.1038/356083a0 1538787

[41] Benkert P Tosatto SC Schomburg DQMEAN. A comprehensive scoring function for model quality assessment. *Proteins*. (2008) 71:261–77. 10.1002/prot.21715 17932912

[42] Duan Y Wu C Chowdhury S Lee MC Xiong G Zhang W A point-charge force field for molecular mechanics simulations of proteins based on condensed-phase quantum mechanical calculations. *J Comput Chem*. (2003) 24:1999–2012. 10.1002/jcc.10349 14531054

[43] Amadei A Linssen AB Berendsen HJ. Essential dynamics of proteins. *Proteins*. (1993) 17:412–25. 10.1002/prot.340170408 8108382

[44] Kumar R Saran S. Comparative modelling unravels the structural features of eukaryotic TCTP implicated in its multifunctional properties: an in silico approach. *J Mol Model*. (2021) 27:20. 10.1007/s00894-020-04630-y 33410974

[45] Doncheva NT Assenov Y Domingues FS Albrecht M. Topological analysis and interactive visualization of biological networks and protein structures. *Nat Protoc*. (2012) 7:670–85. 10.1038/nprot.2012.004 22422314

[46] Shannon P Markiel A Ozier O Baliga NS Wang JT Ramage D Cytoscape: a software environment for integrated models of biomolecular interaction networks. *Genome Res.* (2003) 13:2498–504. 10.1101/gr.1239303 14597658 PMC403769

[47] Trott O Olson AJ. AutoDock Vina: improving the speed and accuracy of docking with a new scoring function, efficient optimization, and multithreading. *J Comput Chem*. (2010) 31:455–61. 10.1002/jcc.21334 19499576 PMC3041641

[48] Alhossary A Handoko SD Mu Y Kwoh CK. Fast, accurate, and reliable molecular docking with QuickVina 2. *Bioinformatics.* (2015) 31:2214–6. 10.1093/bioinformatics/btv082 25717194

[49] Koes DR Baumgartner MP Camacho CJ. Lessons learned in empirical scoring with smina from the CSAR 2011 benchmarking exercise. *J Chem Inf Model.* (2013) 53:1893–904. 10.1021/ci300604z 23379370 PMC3726561

[50] Liu N Xu Z. Using LeDock as a docking tool for computational drug design. *IOP Conf Ser Earth Environ Sci.* (2019) 218:012143. 10.1088/1755-1315/218/1/012143

[51] McNutt AT Francoeur P Aggarwal R Masuda T Meli R Ragoza M GNINA 1.0: molecular docking with deep learning. *J Cheminform.* (2021) 13:43. 10.1186/s13321-021-00522-2 34108002 PMC8191141

[52] Morris GM Huey R Lindstrom W Sanner MF Belew RK Goodsell DS AutoDock4 and AutoDockTools4: automated docking with selective receptor flexibility. *J Comput Chem*. (2009) 30:2785–91. 10.1002/jcc.21256 19399780 PMC2760638

[53] Kumar R Maurya R Saran S. Identification of novel inhibitors of the translationally controlled tumor protein (TCTP): insights from molecular dynamics. *Mol BioSyst*. (2017) 13:510–24. 10.1039/C6MB00850J 28128835

[54] Laskowski RA Swindells MB. LigPlot+: multiple ligand–protein interaction diagrams for drug discovery. *J Chem Inf Model*. (2011) 51:2778–86. 10.1021/ci200227u 21919503

[55] Kumari R Kumar R. Open source drug discovery consortium, Lynn A. g_mmpbsa–a GROMACS tool for high-throughput MM-PBSA calculations. *J Chem Inf Model.* (2014) 54:1951–62. 10.1021/ci500020m 24850022

[56] Bayat A. Science, medicine, and the future: bioinformatics. *BMJ.* (2002) 324:1018–22. 10.1136/bmj.324.7344.1018 11976246 PMC1122955

[57] Andreassi MG Botto N Colombo MG Biagini A Clerico A. Genetic instability and atherosclerosis: can somatic mutations account for the development of cardiovascular diseases? *Environ Mol Mutagen*. (2000) 35:265–9. 10.1002/1098-2280200035:4 10861945

[58] Funnell T Zhang AW Grewal D McKinney S Bashashati A Wang YK Integrated structural variation and point mutation signatures in cancer genomes using correlated topic models. *PLoS Comput Biol*. (2019) 15:e1006799. 10.1371/journal.pcbi.1006799 30794536 PMC6402697

[59] Piattini F Le Foll C Kisielow J Rosenwald E Nielsen P Lutz T A spontaneous leptin receptor point mutation causes obesity and differentially affects leptin signaling in hypothalamic nuclei resulting in metabolic dysfunctions distinct from db/db mice. *Mol Metab*. (2019) 25:131–41. 10.1016/j.molmet.2019.04.010 31076350 PMC6601129

[60] Loos RJ Yeo GS. The bigger picture of FTO-the first GWAS-identified obesity gene. *Nat Rev Endocrinol*. (2014) 10:51–61. 10.1038/nrendo.2013.227 24247219 PMC4188449

[61] Karra E O’Daly OG Choudhury AI Yousseif A Millership S Neary MT A link between FTO, ghrelin, and impaired brain food-cue responsivity. *J Clin Invest.* (2013) 123:3539–51. 10.1172/JCI44403 23867619 PMC3726147

[62] Zhao X Yang Y Sun BF Shi Y Yang X Xiao W FTO-dependent demethylation of N6-methyladenosine regulates mRNA splicing and is required for adipogenesis. *Cell Res.* (2014) 24:1403–19. 10.1038/cr.2014.151 25412662 PMC4260349

[63] Wu R Yao Y Jiang Q Cai M Liu Q Wang Y Epigallocatechin gallate targets FTO and inhibits adipogenesis in an mRNA m^6^A-YTHDF2-dependent manner. *Int J Obes.* (2018) 42:1378–88. 10.1038/s41366-018-0082-5 29795461

[64] Li Z Weng H Su R Weng X Zuo Z Li C FTO plays an oncogenic role in acute myeloid Leukemia as a N^6^-Methyladenosine RNA Demethylase. *Cancer Cell.* (2017) 31:127–41. 10.1016/j.ccell.2016.11.017 28017614 PMC5234852

[65] Niu Y Lin Z Wan A Chen H Liang H Sun L RNA N6-methyladenosine demethylase FTO promotes breast tumor progression through inhibiting BNIP3. *Mol Cancer.* (2019) 18:46. 10.1186/s12943-019-1004-4 30922314 PMC6437932

[66] Zhang C Zhang M Ge S Huang W Lin X Gao J Reduced m6A modification predicts malignant phenotypes and augmented Wnt/PI3K-Akt signaling in gastric cancer. *Cancer Med.* (2019) 8:4766–81. 10.1002/cam4.2360 31243897 PMC6712480

[67] Li J Han Y Zhang H Qian Z Jia W Gao Y The m6A demethylase FTO promotes the growth of lung cancer cells by regulating the m6A level of USP7 mRNA. *Biochem Biophys Res Commun.* (2019) 512:479–85. 10.1016/j.bbrc.2019.03.093 30905413

[68] Zhou S Bai ZL Xia D Zhao ZJ Zhao R Wang YY FTO regulates the chemo-radiotherapy resistance of cervical squamous cell carcinoma (CSCC) by targeting β-catenin through mRNA demethylation. *Mol Carcinog.* (2018) 57:590–7. 10.1002/mc.22782 29315835

[69] Wen W Cho YS Zheng W Dorajoo R Kato N Qi L Meta-analysis identifies common variants associated with body mass index in east Asians. *Nat Genet*. (2012) 44:307–11. 10.1038/ng.1087 22344219 PMC3288728

[70] Okada Y Kubo M Ohmiya H Takahashi A Kumasaka N Hosono N Common variants at CDKAL1 and KLF9 are associated with body mass index in east Asian populations. *Nat Genet*. (2012) 44:302–6. 10.1038/ng.1086 22344221 PMC3838874

[71] Gu Z Bi Y Yuan F Wang R Li D Wang J Polymorphisms are associated with metabolic dysfunction-associated fatty liver disease (MAFLD) susceptibility in the older chinese han population. Clinical interventions in aging. *Clin Interv Aging*. (2020) 15:1333–41. 10.2147/CIA.S254740 32848374 PMC7429205

[72] Ningombam SS Chhungi V Newmei MK Rajkumari S Devi NK Mondal PR Differential distribution and association of FTO rs9939609 gene polymorphism with obesity: a cross-sectional study among two tribal populations of India with East-Asian ancestry. *Gene.* (2018) 647:198–204. 10.1016/j.gene.2018.01.009 29317321

[73] Pereira J Simpkin AJ Hartmann MD Rigden DJ Keegan RM Lupas AN. High-accuracy protein structure prediction in CASP14. *Proteins*. (2021) 89:1687–99. 10.1002/prot.26171 34218458

[74] Varadi M Anyango S Deshpande M Nair S Natassia C Yordanova G AlphaFold Protein Structure Database: massively expanding the structural coverage of protein-sequence space with high-accuracy models. *Nucleic Acids Res.* (2022) 50:D439–44. 10.1093/nar/gkab1061 34791371 PMC8728224

[75] Kumar R. Mutations in passive residues modulate 3D-structure of NDM (New Delhi metallo-β-lactamase) protein that endue in drug resistance: a MD simulation approach. *J Biomol Struct Dyn*. (2021) 26:1–17. 10.1080/07391102.2021.1930165 34034624

[76] Pace CN Fu H Lee Fryar K Landua J Trevino SR Schell D Contribution of hydrogen bonds to protein stability. *Protein Sci.* (2014) 23:652–61. 24591301 10.1002/pro.2449 PMC4005716

[77] Berendsen HJ Hayward S. Collective protein dynamics in relation to function. *Curr Opin Struct Biol*. (2000) 2:165–9. 10.1016/s0959-440x(00)00061-0 10753809

[78] Liu N Pan T. N6-methyladenosine–encoded epitranscriptomics. *Nat Struct Mol Biol*. (2016) 23:98–102. 10.1038/nsmb.3162 26840897

[79] Engel M Eggert C Kaplick PM Eder M Roh S Tietze L The role of m6A/m-RNA methylation in stress response regulation. *Neuron.* (2018) 99:389–403.e9. 10.1016/j.neuron.2018.07.009 30048615 PMC6069762

