# Supplementary material for: Comprehensive mutations analyses of FTO (fat mass and obesity-associated gene) and their effects on FTO’s substrate binding implicated in obesity
Source: Front Nutr. 2022 Jul 18;9:852944. doi: 10.3389/fnut.2022.852944 (PMC9339907; doi:10.3389/fnut.2022.852944)
Supplement: Supplementary file 1 [file Data_Sheet_1.PDF]

Supplementary material

Comprehensive mutations analyses of *FTO* (fat mass and obesity associated gene) and their effects on FTO's substrate binding implicated in obesity

Rakesh Kumar[1§], Somorjit Singh Ningombam[1§], Rahul Kumar[1], Harsh Goel[1], Ajay Gogia[2], Sachin Khurana[2], S.V.S. Deo[3], Sandeep Mathur[4] and Pranay Tanwar[1*]

[1]Laboratory Oncology Unit, Dr.B.R.A.-Institute Rotary Cancer Hospital, All India Institute of Medical Sciences, New Delhi, INDIA-110029

[2]Medical Oncology, Dr.B.R.A.-Institute Rotary Cancer Hospital, All India Institute of Medical Sciences, New Delhi, INDIA-110029

[3]Surgical Oncology, Dr.B.R.A.-Institute Rotary Cancer Hospital, All India Institute of Medical Sciences, New Delhi, INDIA-110029

[4]Department of Pathology, All India Institute of Medical Sciences, New Delhi, INDIA-110029

***Corresponding author:**

Dr. Pranay Tanwar
Laboratory Oncology Unit, Dr.B.R.A.-Institute Rotary Cancer Hospital,
All India Institute of Medical Sciences, New Delhi, INDIA-110029
E-mail: pranaytanwar@gmail.com

Supplementary figures : 10

Supplementary tables : 10

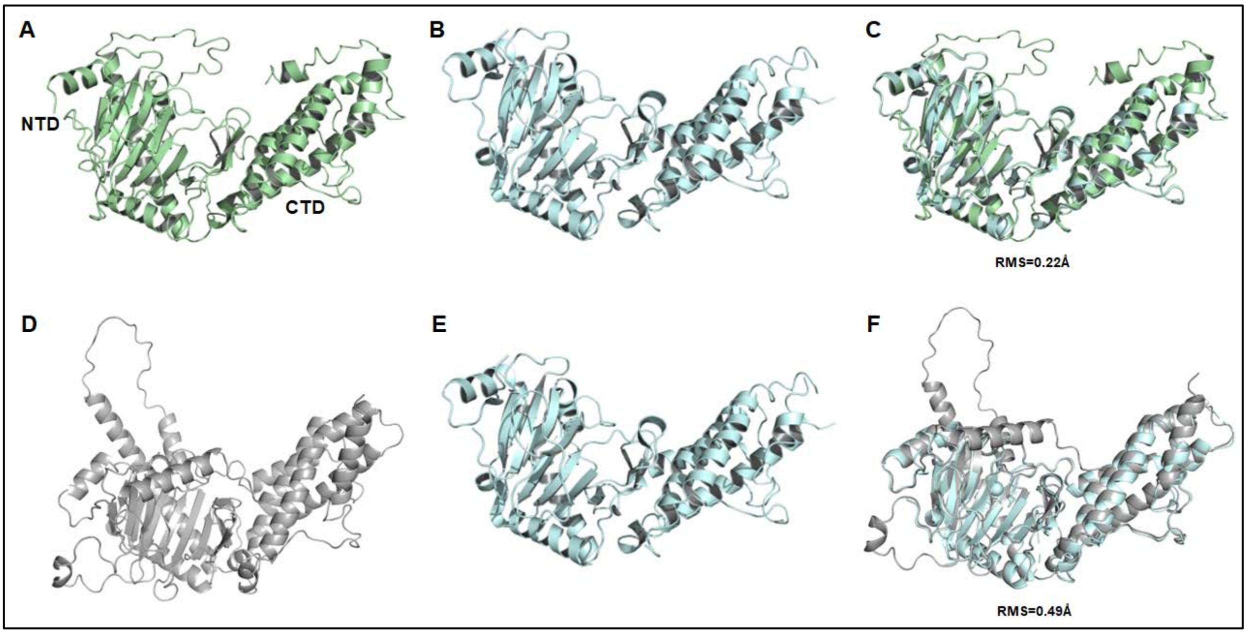

Figure S1. Tertiary model of FTO protein. (A) Full length FTO model from I-TASSER server, (B) FTO structure from PDB, (C) Superposition of I-TASSER model and PDB structure, (D) Full length FTO model from AlphaFold, (E) FTO structure from PDB and (F) Superposition of AlphaFold model and PDB structure. Protein structure were depicted in uniform cartoon mode. [NTD: N-terminal domain and CTD: C-terminal domain].

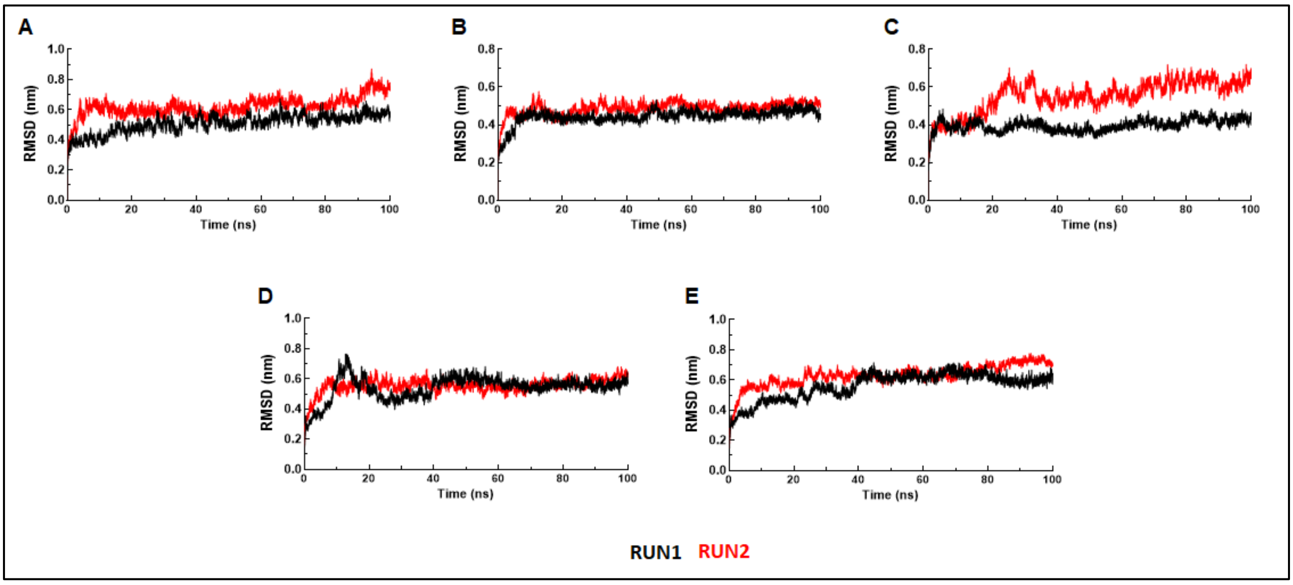

Figure S2. MD simulation profiles of WT and MTs. (A) WT, (B) E234P, (C) R316Q, (D) R322Q and (E) C392D.

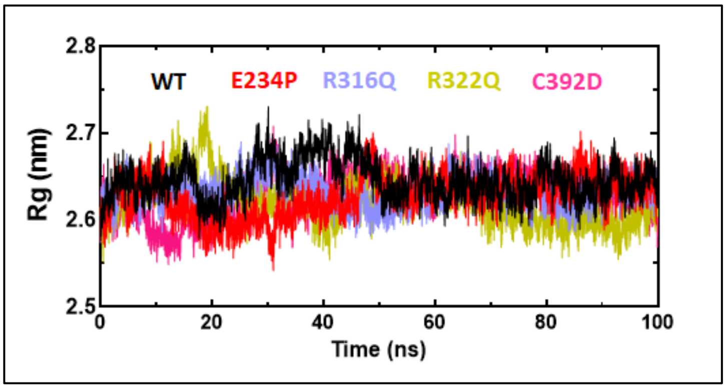

Figure S3. Radius of gyration of WT and MT proteins. WT, E234P, R316Q, R322Q and C392D MTs were labelled in black, red, blue, mustard and pink colour, respectively.

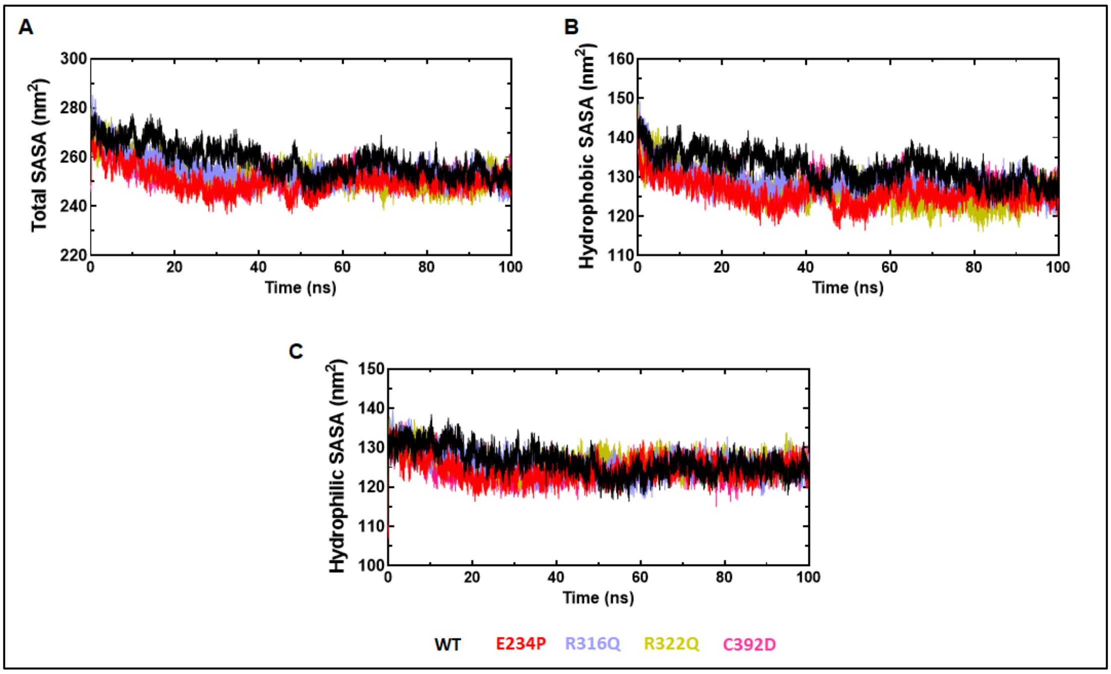

Figure S4. SASA analyses of WT and MTs. (A) Total SASA plot, (B) Hydrophobic SASA plot and (C) Hydrophilic SASA plot.

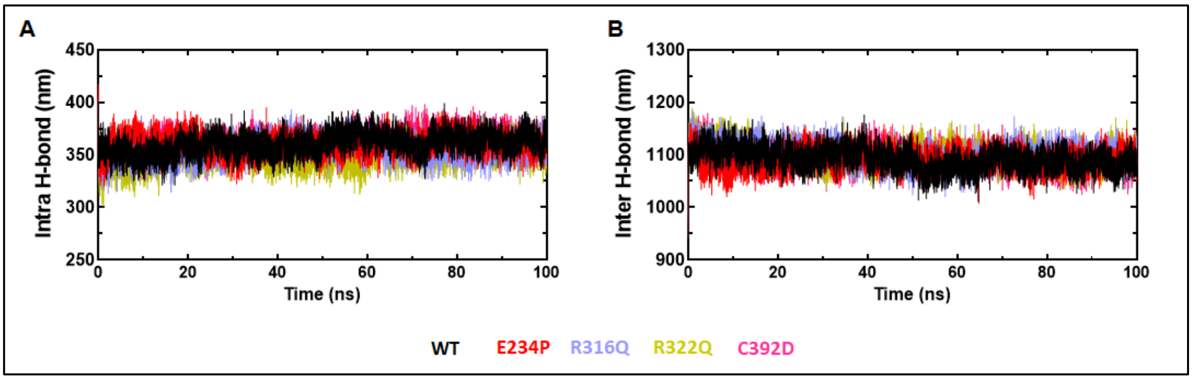

Figure S5. Hydrogen bond analyses of WT and MTs. (A) Intra or protein-protein H-bond plots and (B) Inter or protein-water H-bond plots.

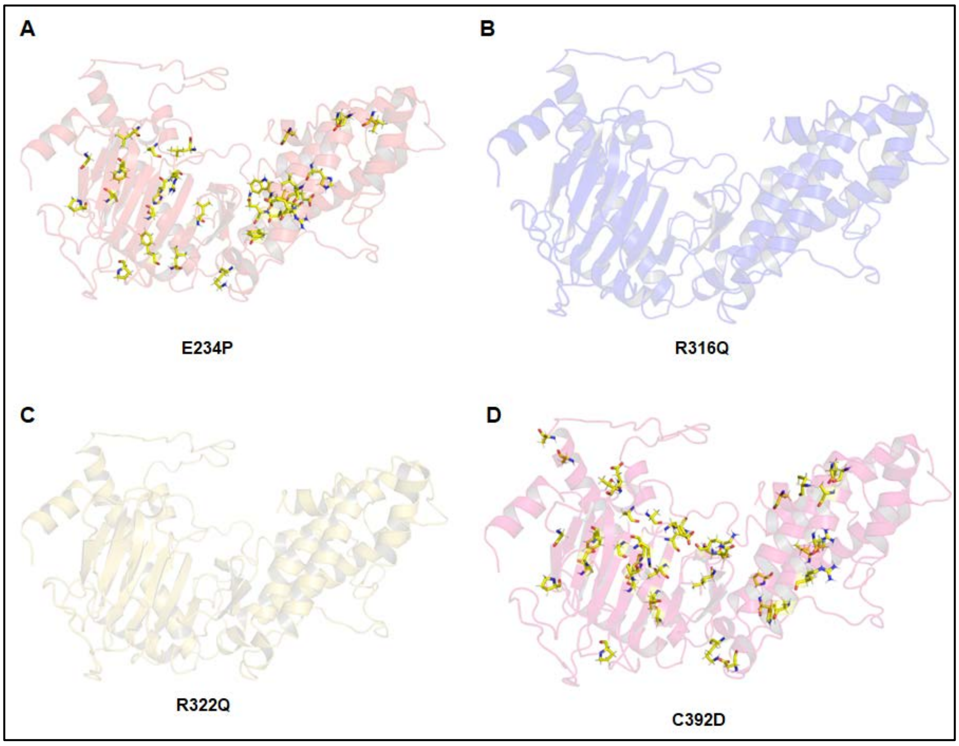

Figure S6. Betweenness centrality (C_B^{WT}-C_B^{MT}≥0.02) analysis. (A) E234P, (B) R316Q, (C) R322Q and (D) C392D. Residues having C_B values ≥0.02 were displayed in stick mode with yellow colour and MT proteins were represented in uniform colour cartoon mode.

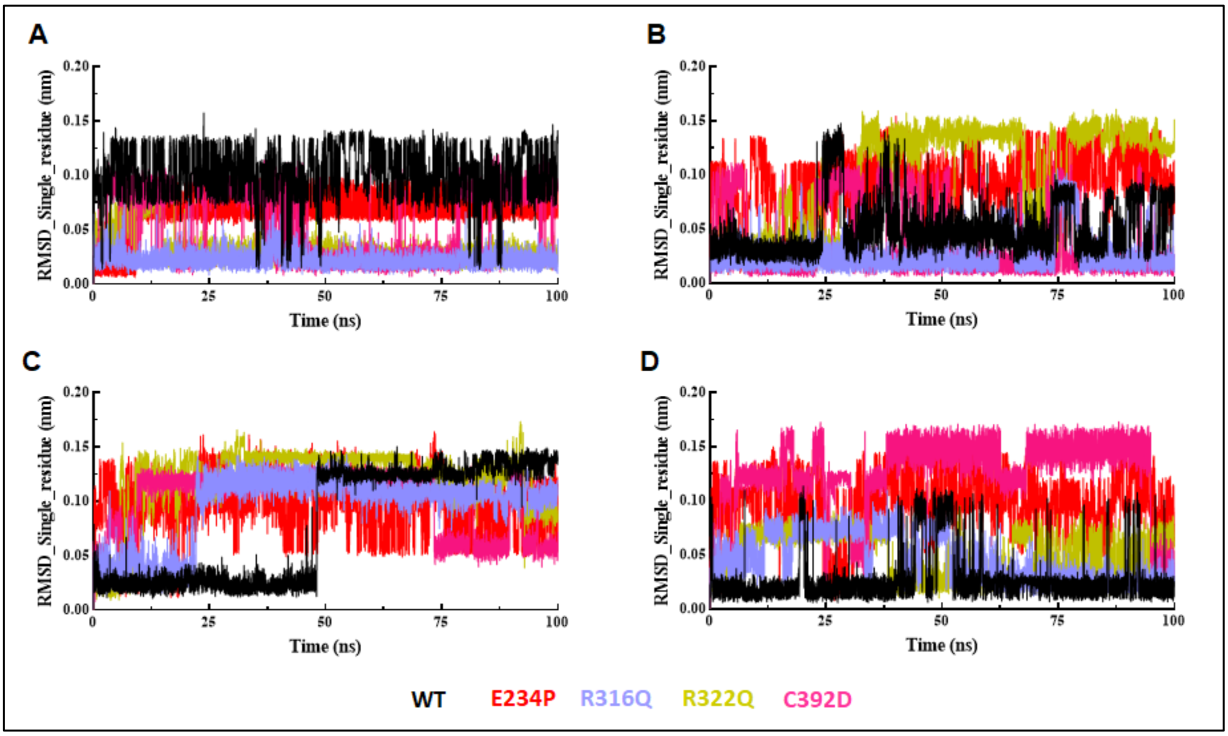

Figure S7. MD simulation profile of individual mutant residues. RMSD plots of E234P (A), R316Q (B), R322Q (C) and C392D (D).

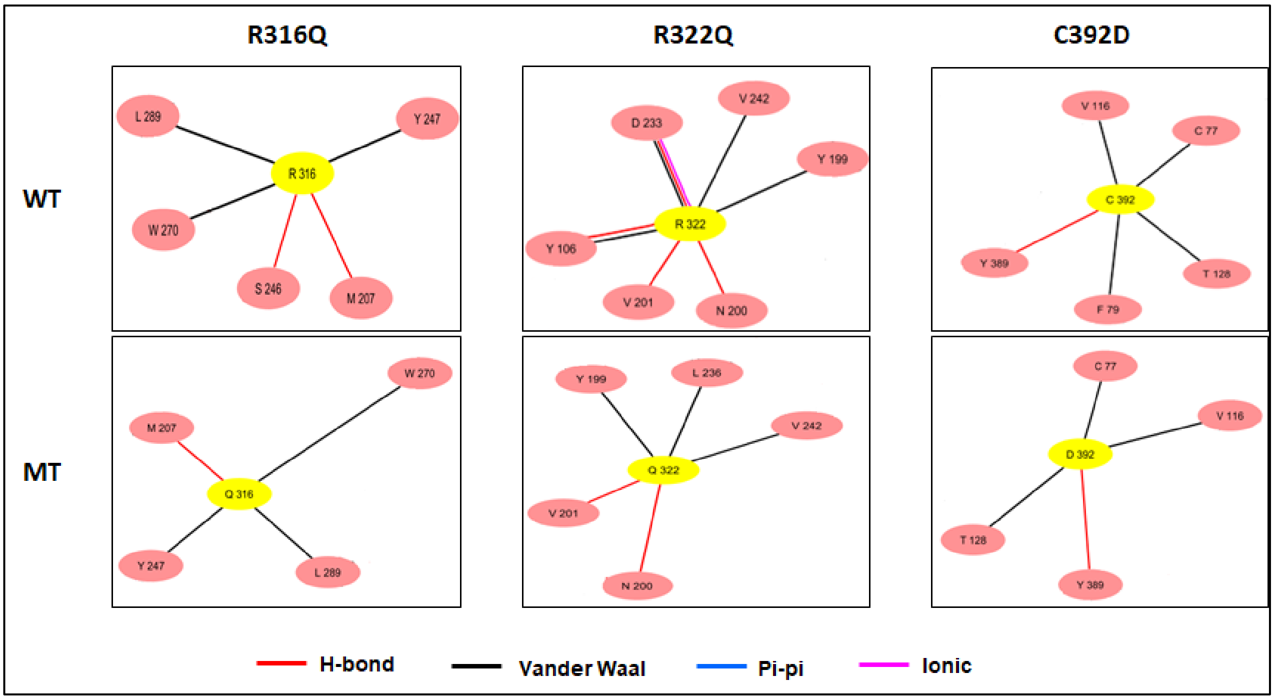

Figure S8. Residues interaction maps of R316Q, R322Q and C392D MTs. Mutated residues were highlighted in yellow colour.

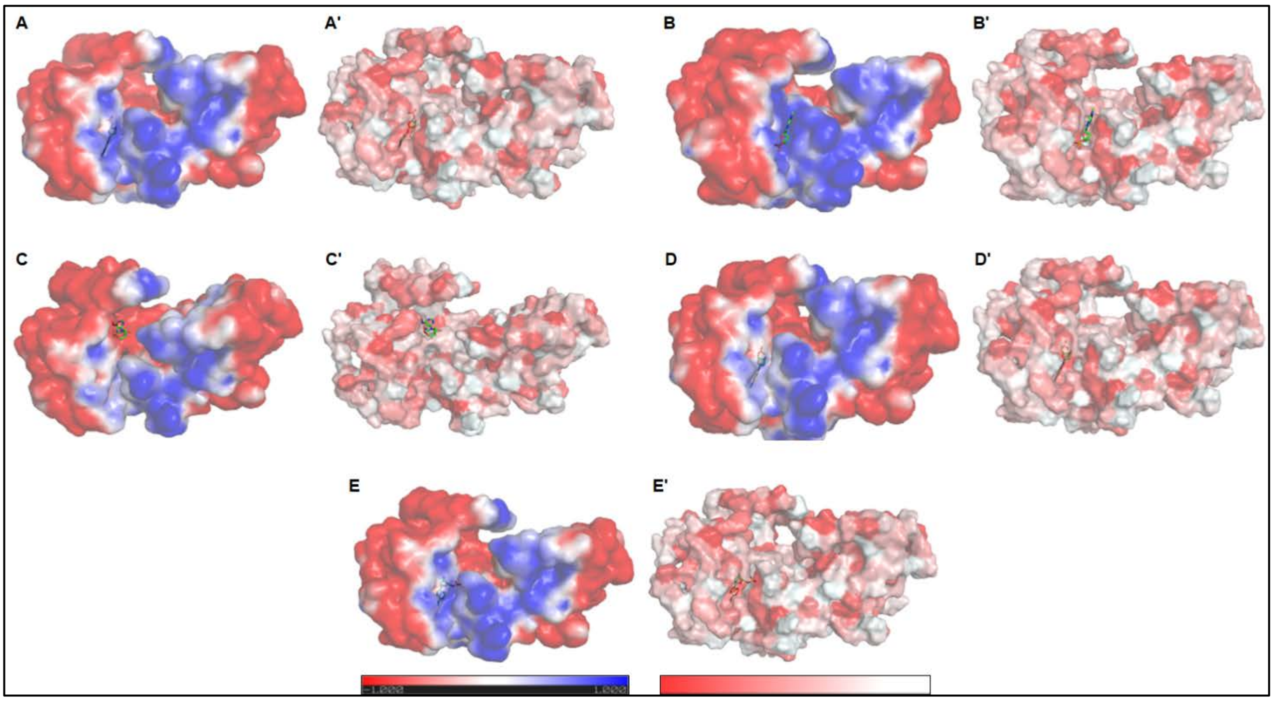

Figure S9. Electrostatic potential and Hydrophobicity maps. Electrostatic maps of (A) E234P, (B) R316Q, (C) R322Q and (D) C392D. Figure panels A' - E' showed hydrophobicity maps of WT and all MTs. Ligands were displayed in stick mode. Red and blue colour in electrostatic map of protein represented negative and positive charged surfaces while red and white colour in hydrophobic map of protein represented intensity of hydrophobicity.

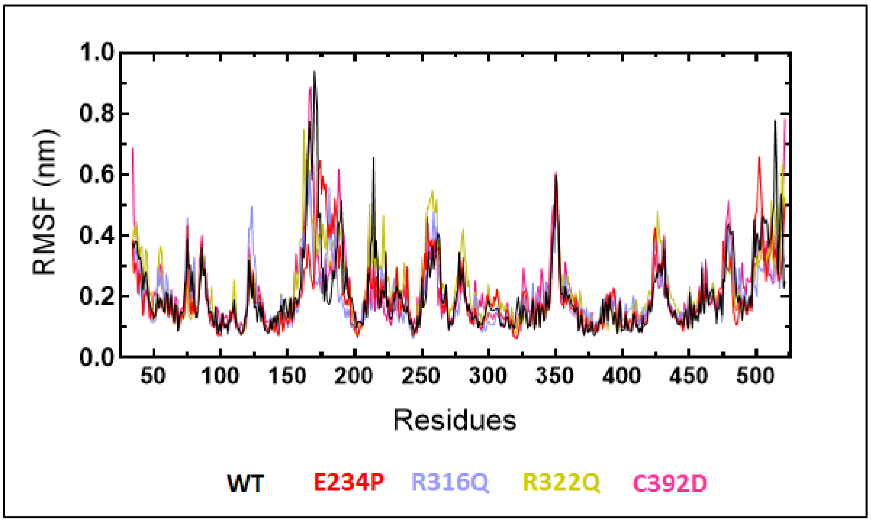

Figure S10. Root mean square fluctuations of WT– and MT-6mA complexes.

Table S1. Functional consequences of FTO variants predicted by PredictSNP server

Sl.no	Variants	Predict SNP	MAPP	PhD-SNP	Polyphen-1	Polyphen-2	SIFT	SNAP	PANTHER
**1**	**K88R**	74 N	63 N	83 N	67 N	40 D	73 N	58 N	71 N
**2**	**G182A**	74 N	66 N	83 N	67 N	40 D	71 N	55 N	67 N
**3**	**A134T**	73 N	43 D	72 N	67 N	40 D	79 N	67 N	64 N
**4**	**SR316G**	79 D	84 D	51 N	74 D	81 D	79 N	67 N	64 N
**5**	**S2526N**	83 N	75 N	83 N	67 N	68 N	71 N	61 N	55 N
**6**	**A163T**	83 N	65 N	83 N	67 N	70 N	78 N	61 N	64 N
**7**	**E325V**	65 N	68 N	58 N	67 N	40 D	75 N	56 N	48 N
**8**	**S319F**	87 D	77 D	61 D	74 D	81 D	79 D	85 D	61 D
**9**	**H261Q**	83 N	77 D	83 N	67 N	72 N	81 N	50 N	71 N
**10**	**T320I**	61 D	88 D	55 N	67 N	68 D	79 D	81 D	55 N
**11**	**T436P**	55 D	59 D	66 N	59 D	47 D	73 N	72 D	62 N
**12**	**K88R**	74 N	65 N	83 N	67 N	40 D	68 N	58 N	71 N
**13**	**D36E**	75 N	77 N	89 N	67 N	40 D	81 N	61 N	68 N
**14**	**S260C**	63 N	75 N	83 N	74 D	56 D	65 N	50 N	57 D
**15**	**W383L**	87 D	77 D	59 D	74 D	81 D	53 D	81 D	52 N
**16**	**D89E**	60 N	57 D	66 N	59 D	56 D	73 N	58 N	56 N
**17**	**I492T**	74 N	72 N	58 D	67 N	76 N	67 N	50 N	64 N
**18**	**M207L**	61 D	76 D	59 D	67 N	60 D	79 D	50 N	67 N
**19**	**R445H**	51 D	64 N	51 N	74 D	81 D	46 D	55 N	57 D
**20**	**R96P**	87 D	88 D	82 D	74 D	81 D	79 D	85 D	65 D
**21**	**Q314K**	83 N	64 N	68 N	67 N	71 N	71 N	61 N	69 N
**22**	**R80N**	76 D	56 D	58 N	74 D	81 D	54 D	72 D	74 D
**23**	**N432S**	83 N	76 N	83 N	67 N	72 N	79 N	55 N	75 N
**24**	**I132T**	60 N	57 D	66 N	59 D	40 D	71 N	50 N	49 N
**25**	**I148M**	83 N	73 N	83 N	67 N	71 N	67 N	67 N	65 N
**26**	**G251D**	74 N	72 N	89 N	67 N	73 N	76 N	63 D	49 N
**27**	**N344D**	55 D	57 D	78 N	59 D	65 D	43 D	58 N	62 N

**28**	**R80Q**	83 N	76 N	89 N	67 N	61 N	48 N	55 N	48 N
**29**	**E433Q**	64 D	41 D	78 N	59 D	47 D	45 D	72 D	56 N
**30**	**N143S**	76 D	57 D	45 N	59 D	65 D	53 D	56 D	52 N
**31**	**L262P**	74 N	64 N	72 N	67 N	71 N	45 D	50 N	49 N
**32**	**I50L**	83 N	70 N	83 N	67 N	72 N	73 N	71 N	70 N
**33**	**L28V**	75 N	65 N	72 N	67 N	60 D	67 N	58 N	67 N
**34**	**V201I**	63 N	79 N	72 N	59 D	71 N	46 D	55 N	67 N
**35**	**P93R**	61 D	86 D	66 N	74 D	59 D	76 N	81 D	49 N
**36**	**L146M**	75 N	64 N	83 N	59 D	55 D	66 N	59 N	56 N
**37**	**A311S**	63 N	59 D	51 N	67 N	56 D	68 N	67 N	64 N
**38**	**R322G**	87 D	84 D	58 D	54 D	44 D	53 D	81 D	47 N
**39**	**E263K**	71 N	41 D	72 N	67 N	41 D	76 N	50 N	52 N
**40**	**D114N**	74 N	75 N	83 N	67 N	55 D	70 N	55 N	68 N
**41**	**I125R**	60 N	65 N	72 N	74 D	59 D	77 N	72 D	65 N
**42**	**K2Q**	60 N	80 N	83 N	59 D	59 D	79 D	61 N	-
**43**	**I97V**	83 N	74 N	72 N	67 N	70 N	76 N	67 N	71 N
**44**	**D258G**	65 N	41 D	83 N	67 N	70 N	54 N	62 D	58 N
**45**	**K45N**	74 N	76 N	78 N	67 N	55 D	75 N	55 N	62 N
**46**	**A286V**	63 N	43 D	72 N	59 D	55 D	81 N	61 N	68 N
**47**	**L489F**	61 D	77 D	83 N	74 D	50 D	79 D	58 N	56 N
**48**	**L51I**	65 N	80 N	83 N	59 D	50 D	65 N	67 N	65 N
**49**	**K160R**	83 N	73 N	89 N	67 N	72 N	76 N	61 N	69 N
**50**	**P399L**	55 D	56 D	58 N	74 D	72 N	53 D	56 D	57 D
**51**	**T32A**	75 N	62 D	78 N	67 N	70 N	61 N	55 N	68 N
**52**	**P288L**	87 D	86 D	68 D	74 D	81 D	79 D	56 D	47 N
**53**	**K126R**	75 N	77 N	89 N	67 N	81 D	71 N	61 N	67 N
**54**	**A286V**	60 N	43 D	72 N	59 D	55 D	81 N	61 N	68 N
**55**	**K478V**	74 N	43 D	83 N	67 N	81 D	77 N	58 N	62 N
**56**	**A286T**	74 N	43 D	78 N	67 N	70 N	76 N	71 N	75 N
**57**	**M1L**	75 N	76 N	89 N	67 N	76 N	79 D	58 N	-
**58**	**I269V**	83 N	80 N	78 N	67 N	76 N	90 N	71 N	71 N
**59**	**Q385P**	87 D	82 D	86 D	74 D	81 D	79 D	81 D	57 D
**60**	**C308R**	87 D	88 D	77 D	74 D	59 D	79 D	81 D	66 D
**61**	**T6I**	51 D	77 N	78 N	59 D	59 D	53 D	56 D	52 N
**62**	**H62R**	76 D	77 D	51 N	74 D	50 D	59 D	56 D	47 N
**63**	**A68G**	74 N	70 N	72 N	67 N	50 D	70 N	58 N	39 N
**64**	**D25G**	55 D	64 N	66 N	59 D	50 D	53 D	62 D	56 N
**65**	**R10Q**	60 N	77 N	66N	59 D	65 D	53 D	55 N	48 N
**66**	**R322Q**	64 D	77 D	45 N	74 D	81 D	79 D	50 N	55 N
**67**	**A130P**	83 N	73 N	78 N	67 N	76 N	78 N	55 N	48 N
**68**	**L298I**	75 N	73 N	78 N	67 N	81 D	68 N	55 N	-
**69**	**W383R**	87 D	72 D	68 D	74 D	81 D	53 D	85 D	66 D

**70**	**L44V**	74 N	65 N	83 N	67 N	50 D	76 N	61 N	65 N
**71**	**D467Y**	72 D	46 D	58 D	74 D	50 D	79 D	62 D	49 N
**72**	**V421F**	63 N	56 D	58 N	74 D	72 N	66 N	50 N	49 N
**73**	**L70F**	74 N	77 N	68 N	67 N	72 N	45 D	61 N	48 N
**74**	**L496P**	76 D	77 D	77 D	74 D	68 N	79 D	81 D	66 D
**75**	**E156K**	83 N	76 N	83 N	67 N	68 N	76 N	61 N	65 N
**76**	**T4P**	52 D	59 D	83 N	59 D	47 D	45 D	50 N	57 N
**77**	**E129K**	83 N	66 N	72 N	67 N	70 N	76 N	50 N	62 N
**78**	**T150I**	87 D	41 D	58 D	74 D	50 D	53 D	56 D	68 N
**79**	**R84S**	75 N	65 N	68 N	67 N	-	67 N	56 D	57 D
**80**	**E60D**	74 N	74 N	89 N	67 N	50 D	76 N	67 N	67 N
**81**	**A241V**	60 N	56 D	78 N	67 N	65 D	46 D	61 N	56 N
**82**	**R455M**	65 D	66 D	83 N	74 D	65 D	79 D	85 D	66 D
**83**	**L170W**	60 N	64 N	78 N	74 N	65 D	43 D	50 N	67 D
**84**	**A341S**	61 D	59 D	72 N	74 D	68 D	53 D	58 N	64 N
**85**	**R52Q**	63 N	72 N	78 N	59 D	68 D	75 N	55 N	48 N
**86**	**R265G**	60 N	75 D	72 N	67 N	63 N	46 D	62 D	48 N
**87**	**P169S**	83 N	77 N	72 N	67 N	63 N	76 N	50 N	64 N
**88**	**T26I**	60 N	65 N	66 N	59 D	55 D	43 D	50 N	56 N
**89**	**V272A**	60 N	77 D	58 N	67 N	55 D	43 D	55 N	64 N
**90**	**R96C**	72 D	91 D	82 D	74 D	79 N	79 D	85 D	72 D
**91**	**E129D**	83 N	77 N	89 N	67 N	79 N	76 N	57 N	71 N
**92**	**V272A**	60 N	77 D	58 N	67 N	55 D	43 D	55 N	64 N
**93**	**T26I**	60 N	65 N	66 N	59 D	55 D	43 D	50 N	56 N
**94**	**K2Q**	60 N	80 N	83 N	59 D	68 D	79 D	61 N	63 N
**95**	**Q385P**	87 D	82 D	86 D	74 D	81 D	79 D	81 D	57 D
**96**	**G264D**	60 N	48 N	72 N	67 N	54 D	68 N	62 D	66 D
**97**	**K194R**	83 N	63 N	83 N	67 N	74 N	76 N	61 N	67 N
**98**	**P117S**	87 D	86 N	59 D	74 D	81 D	79 D	62 D	57 D
**99**	**E253K**	83 N	75 N	83 N	67 N	72 N	77 N	55 N	71 N
**100**	**P47S**	74 N	79 N	89 N	67 N	74 N	90 N	56 D	65 N
**101**	**T115M**	76 D	62 N	55 N	74 D	81 D	79 D	56 D	65 D
**102**	**P465L**	63 N	86 N	58 N	67 N	45 D	65 N	50 N	47 N
**103**	**G251D**	74 N	72 N	89 N	67 N	73 N	76 N	62 D	49 N
**104**	**I151M**	63 N	70 N	78 N	74 D	45 D	66 N	71 N	56 N
**105**	**D81G**	87 D	75 N	68 D	74 D	81 D	53 D	85 D	47 N
**106**	**R3C**	61 D	41 N	58 N	74 D	74 D	79 D	56 D	72 D
**107**	**Q402H**	74 N	75 N	89 N	67 N	63 D	70 N	67 N	69 N
**108**	**K48Q**	63 N	73 N	72 N	74 D	63 D	71 N	61 N	62 N
**109**	**T304A**	61 D	77 N	58 D	59 D	81 D	68 N	50 N	65 N
**110**	**W42C**	87 D	46 N	82 D	74 D	81 D	45 D	81 D	74 D
**111**	**L109V**	61 D	66 N	55 N	59 D	81 D	46 D	58 N	56 B

**112**	**Y36C**	51 D	70 N	45 N	74 D	56 D	71 N	72 D	65 D
**113**	**C77R**	87 D	46 N	88 D	74 D	56 D	46 D	81 D	70 D
**114**	**K74X**	60 N	66 N	58 N	59 D	47 D	45 D	58 N	56 N
**115**	**K45E**	65 N	59 N	83 M	67 N	47 D	73 N	50 N	65 N
**116**	**T71A**	74 N	59 N	68 N	67 N	72 N	75 N	61 N	62 N
**117**	**E471G**	65 N	48 N	78 N	59 D	72 N	75 N	50 N	65 N
**118**	**T71I**	63 N	57 N	45 N	67 N	65 D	53 N	50 N	38 N
**119**	**T330I**	97 D	77 N	68 D	74 D	65 D	79 D	81 D	55 N
**120**	**V83E**	87 D	92 N	73 N	74 D	81 D	79 D	72 D	66 D
**121**	**L44R**	65 N	41 N	55 N	67 N	69 N	78 N	62 D	56 N
**122**	**K16R**	60N	65 N	72 N	59 D	61 N	79 D	56 D	64 N
**123**	**V120M**	83 N	66 N	78 N	67 N	61 N	70 N	61 N	49 N
**124**	**I99V**	74 N	41 N	72 N	67 N	73 N	77 N	67 N	71 N
**125**	**T150A**	74 N	46 N	72 N	67 N	73 N	73 N	55 N	68 N
**126**	**P252R**	60 N	64 N	78 N	67 N	54 N	46 D	56 D	56 N
**127**	**P399A**	63 N	64 N	72 N	67 N	54 N	66 N	62 D	52 N
**128**	**G76D**	61 D	56 N	88 D	67 N	50 D	67 N	85 D	66 D
**129**	**P315L**	65 D	41 N	66 N	59 D	50 D	45 D	56 D	57 D
**130**	**P288T**	87 D	86 N	59 D	74 D	81 D	79 D	58 D	48 N
**131**	**R316W**	87 D	77 N	68 D	59 D	81 D	79 D	81 D	72 D
**132**	**D299H**	76 D	59 N	51 N	74 D	60 D	53 D	72 D	61 D
**133**	**R473Q**	63 N	74 N	83 N	59 D	68 D	76 N	50 N	56 N
**134**	**E281D**	83 N	72 N	78 N	67 N	76 N	76 N	61 N	65 N
**135**	**H73R**	83 N	80 N	89 N	67 N	76 N	77 N	61 N	68 N
**136**	**Q86P**	51 D	75 N	68 D	67 N	47 D	68 N	50 N	48 N
**137**	**Q43H**	74 N	63 N	78 N	67 N	47 D	61 N	55 N	52 N
**138**	**E64G**	65 N	41 N	78 N	67 N	68 N	74 N	62 D	56 N
**139**	**E8K**	75 N	68 N	78 N	67 N	68 N	79 D	50 N	65 N
**140**	**C326R**	61 D	59 N	82 D	74 D	72 N	53 N	89 D	66 D
**141**	**S56R**	74 N	41 N	72 N	67 N	72 N	71 N	50 N	49 N
**142**	**R388P**	87 D	78 N	77 D	74 D	81 D	53 D	72 D	61 D
**143**	**R462L**	75 N	70 N	68 N	67 N	81 D	75 N	50 N	49 N
**144**	**N387D**	75 N	72 N	89 N	67 N	65 D	76 N	55 N	70 N
**145**	**F38L**	76 D	43 N	58 D	59 D	65 D	56 D	50 N	57 N
**146**	**G122W**	87 D	51 N	58 D	74 D	81 D	79 D	72 D	77 D
**147**	**H271R**	83 N	73 N	66 N	67 N	70 N	77 N	58 N	67 N
**148**	**T6A**	65 N	65 N	83 N	67 N	55 D	45 D	55 N	65 N
**149**	**H127Y**	83 N	76 N	78 N	67 N	87 N	90 N	55 N	67 N
**150**	**G273A**	68 N	46 D	58 N	67 N	41 D	78 N	67 N	56 N
**151**	**K45R**	83 N	70 N	89 N	67 N	68 N	79 N	61 N	67 N
**152**	**E67D**	83 N	68 N	78 N	67 N	72 N	71 N	71 N	67 N
**153**	**K2N**	60 N	76 N	89 N	59 D	59 D	79 D	58 N	57 N

**154**	**R390G**	74 N	70 N	72 N	67 N	74 N	76 N	62 D	63 N
**155**	**A14T**	60 N	75 N	78 N	59 D	83 D	53 D	61 N	64 N
**156**	**P267T**	74 N	70 N	72 N	67 N	50 D	66 N	50 N	62 N
**157**	**K45Q**	65 N	72 N	83 N	59 D	56 D	76 N	55 N	63 N
**158**	**P5R**	60 N	82 D	89 N	59 D	40 D	66 N	50 N	71 N
**159**	**E9Q**	63 N	78 N	83 N	59 D	59 D	68 N	50 N	48 N
**160**	**F139L**	83 N	72 N	78 N	67 N	76 N	87 N	61 N	71 N
**161**	**P117R**	87 D	88 D	68 D	74 D	81 D	79 D	85 D	65 D
**162**	**R3P**	61 D	41 D	72 N	59 D	65 D	79 D	50 N	57 D
**163**	**D268G**	83 N	73 N	72 N	67 N	70 N	77 N	50 NN	56 N
**164**	**D480N**	68 N	41 D	83 N	67 N	50 D	70 N	50 N	56 N
**165**	**H290P**	60 N	46 D	61 D	67 N	71 N	77 N	56 D	57 D
**166**	**V57L**	83 N	71 N	89 N	67 N	73 N	89 N	71 N	71 N
**167**	**N124H**	52 D	70 N	78 N	74 D	59 D	46 D	56 D	67 N
**168**	**T111N**	61 D	77 D	68 D	74 D	63 D	71 N	58 N	57 D
**169**	**I438V**	75 N	57 D	83 N	67 N	74 N	76 N	67 N	75 N
**170**	**V237A**	63 N	59 D	55 N	67 N	55 D	67 N	58 N	55 N
**171**	**T23I**	76 D	66 D	55 N	74 D	68 D	53 D	62 D	48 N
**172**	**G103D**	87 D	88 D	88 D	74 D	81 D	79 D	85 N	66 D
**173**	**M297L**	63 N	77 D	55 N	67 N	47 D	70 N	50 N	71 N
**174**	**L23V**	74 N	64 N	72 N	67 N	55 D	71 N	61 N	56 N
**175**	**I139V**	83 N	77 N	78 N	67 N	87 N	79 N	71 N	71 N
**176**	**N164H**	74 N	76 N	89 N	67 N	70 N	45 D	55 N	48 N
**177**	**D36V**	52 D	43 D	68 N	59 D	50 N	66 N	56 D	47 N
**178**	**F296S**	61 D	62 D	66 N	67 N	41 D	79 D	72 D	48 N
**179**	**D293V**	87 D	57 D	68 D	74 D	81 D	79 D	85 D	57 D
**180**	**R178T**	75 N	76 N	83 N	67 N	64 N	68 N	62 D	67 N
**181**	**V179L**	83 N	64 N	89 N	67 N	68 D	77 N	61 N	71 N
**182**	**H75Q**	74 N	77 N	68 N	59 D	87 N	71 N	50 N	61 D
**183**	**Y295C**	87 D	82 D	68 D	74 D	40 D	79 D	89 D	69 D
**184**	**L301F**	73 N	41 D	78 N	67 N	40 D	78 N	67 N	55 N
**185**	**M287I**	63 N	77 D	55 N	67 N	40 D	61 N	50 N	67 N
**186**	**L301F**	73 N	41 D	78 N	67 N	40 D	78 N	67 N	55 N
**197**	**Y30H**	76 D	43 D	68 D	74 D	68 D	79 D	50 N	61 D
**188**	**P47R**	63 N	65 N	72 N	67 N	68 D	76 N	62 D	49 N
**189**	**I287T**	65 N	43 D	58 N	67 N	55 D	70 N	55 N	56 N
**190**	**C472Y**	52 D	70 N	59 D	74 D	55 D	90 N	62 D	68 D
**191**	**K162R**	83 N	79 N	83 N	67 N	72 N	76 N	61 N	68 N
**192**	**P33T**	61 D	48 D	51 N	74 D	72 N	79 D	56 D	66 D
**193**	**T26A**	68 N	43 D	83 N	67 N	50 D	71 N	61 N	68 N
**194**	**V65I**	74 N	70 N	55 N	67 N	50 D	68 N	61 N	67 N
**195**	**I151T**	83 N	77 N	68 N	67 N	72 N	76 N	55 N	64 N

**196**	**H307Y**	72 D	84 D	73 D	74 D	72 N	79 D	62 D	52 N
**197**	**N164K**	83 N	70 N	72 N	67 N	69 N	76 N	55 N	63 N
**198**	**Q306K**	76 D	57 D	51 N	59 D	63 D	53 D	56 D	64 N
**199**	**I99T**	76 D	58 D	51 N	59 N	63 D	53 D	56 D	49 N
**200**	**L157V**	75 N	63 D	72 N	67 N	-	77 N	71 N	64 N
**201**	**F67L**	63 N	57 D	51 N	67 N	54 D	66 N	58 N	71 N
**202**	**F317I**	61 D	62 D	61 D	67 N	50 D	79 D	50 N	56 N
**203**	**L146R**	87 D	91 D	86 D	74 D	50 D	79 D	81 D	66 D
**204**	**E11D**	65 N	68 N	83 N	67 N	54 D	45 D	58 N	56 N
**205**	**R112T**	87 D	86 D	88 D	74 D	81 D	79 D	81 D	57 D
**206**	**L157P**	63 N	92 D	59 D	67 N	68 N	74 N	55 N	57 D
**207**	**E451Q**	65 N	72 N	66 N	67 N	54 D	53 D	58 N	56 N
**208**	**I85F**	63 N	72 N	68 N	74 D	54 D	67 N	67 N	48 N
**209**	**L82P**	87 D	72 D	77 D	74 D	54 D	53 D	81 D	70 D
**210**	**D394G**	60 N	65 N	68 N	59 D	55 D	53 N	62 D	49 N
**211**	**P5S**	83 N	85 N	83 N	67 N	61 N	73 N	55 N	75 N
**212**	**D479H**	55 D	70 N	72 N	74 D	65 D	43 D	62 D	52 N
**213**	**Q86H**	60 N	46 D	68 N	59 D	65 D	67 N	58 N	55 N
**214**	**T4I**	83 N	72 N	78 N	67 N	76 N	71 N	50 N	70 N
**215**	**S313L**	83 N	66 N	83 N	67 N	76 N	79 N	55 N	71 N
**216**	**C392R**	61 D	48 D	51 N	74 D	59 D	75 N	85 D	66 D
**217**	**P119L**	60 N	41 D	68 N	59 D	59 D	53 N	50 N	56 N
**218**	**F176I**	83 N	79 N	66 N	67 N	61 N	68 N	58 N	67 N
**219**	**P267H**	65 N	70 N	68 N	67 N	50 D	61 N	56 D	49 N
**220**	**C294Y**	87 D	57 D	68 D	74 D	81 D	79 D	72 D	66 D
**221**	**Y106H**	87 D	81 D	68 D	74 D	59 D	79 D	85 D	61 D
**222**	**H261Y**	61 N	46 D	83 N	67 N	40 D	43 D	55 N	67 N
**223**	**R432S**	68 N	74 N	78 N	67 N	40 D	76 N	62 D	63 N
**224**	**K16N**	65 D	63 D	72 N	59 D	59 D	49 D	56 D	56 N
**225**	**S58Y**	61 D	63 D	83 N	74 D	59 D	79 D	58 N	49 N
**226**	**G312S**	87 D	57 D	68 D	59 D	81 D	79 D	56 D	49 N
**227**	**W395R**	76 D	86 D	51 N	74 D	68 D	79 D	85 N	66 D
**228**	**V94I**	51 D	77 D	78 N	67 N	60 D	79 D	55 N	67 N
**229**	**G412R**	74 N	65 N	78 N	67 N	74 D	76 N	56 D	71 N
**230**	**N302S**	61 D	75 D	55 N	67 N	56 D	79 D	56 D	56 N
**231**	**A133V**	65 N	56 D	78 N	67 N	56 D	77 N	67 N	57 N
**232**	**Q381E**	87 D	82 D	61 D	59 D	60 D	53 D	56 D	64 N
**233**	**W396G**	87 D	82 D	77 D	74 D	41 D	79 D	85 D	61 D
**234**	**C397W**	55 D	41 D	66 N	74 D	59 D	68 N	62 D	69 D
**235**	**C397G**	63 N	64 N	68 N	67 D	59 D	76 N	56 D	56 N
**236**	**T436P**	55 D	59 D	66 N	59 D	63 N	73 N	72 D	62 N
**237**	**T436P**	55 D	59 D	66 N	59 D	63 D	73 N	72 D	62 N

**238**	**R449K**	63 N	56 D	78 N	67 N	63 D	68 N	50 N	67 N
**239**	**R96M**	79 D	81 D	51 N	74 D	81 D	79 D	85 D	69 D
**240**	**Y108A**	87 D	88 D	68 D	74 D	81 D	79 D	85 D	61 D
**241**	**C392D**	61 D	48 D	58 N	59 D	65 D	71 N	85 D	65 D
**242**	**E234P**	76 D	77 D	51 N	74 D	65 D	46 D	72 D	66 D
**243**	**R316Q**	76 D	77 D	55 N	74 D	81 D	79 D	62 D	55 N
**244**	**F114D**	87 D	92 D	88 D	74 D	81 D	79 D	85 D	68 D
**245**	**R322Q**	64 D	77 D	45 N	74 D	81 D	79 D	50 N	55 N

N=Neutral; D=Deleterious

Table S2. List of variants showed high pathogenicity

S. No	Variants	Predict SNP	MAPP	PhD-SNP	PolyPhen-1	PolyPhen-2	SIFT	SNAP	PANTHER
1	**R96M**	79 D	81 D	51 N	74 D	81 D	79 D	85 D	69 D
2	**Y108A**	87 D	88 D	68 D	74 D	81 D	79 D	85 D	61 D
3	**F114D**	87 D	92 D	88 D	74 D	81 D	79 D	85 D	68 D
4	**E234P**	76 D	77 D	51 N	74 D	65 D	46 D	72 D	66 D
5	**R316Q**	76 D	77 D	55 N	74 D	81 D	79 D	62 D	55 N
6	**R322Q**	64 D	77 D	45 N	74 D	81 D	79 D	50 N	55 N
7	**C392D**	61 D	48 D	58 N	59 D	65 D	71 N	85 D	65 D

N=Neutral; D=Deleterious

Table S3. Evolutionary conservation analysis.

Variants	Protein Residue Conservation Prediction
**E234P**	0.807
**R316Q**	0.822
**R322Q**	0.813
**C392D**	0.839

Table S4. Structure validation of WT and MT proteins

Variants	Favoured	Allowed	Disallowed	ProSA (Z-score)	ERRAT	QMEAN (Z-score)
**WT**	73.8%	24.8%	1.4%	-10.04	85.833	-5.35
**E234P**	73.8%	24.8%	1.4%	-10.18	85.83	-5.38
**R316Q**	73.8%	24.8%	1.4%	-10.01	86.46	-5.34
**R322Q**	73.8%	24.8%	1.4%	-10.04	86.67	-5.28
**C392D**	73.8%	24.8%	1.4%	-9.9	85.83	-5.34

Table S5. Secondary structure formation of WT and MT proteins

Variants	Structure	Coil	β-Sheet	β-Bridge	Bend	Turn	α-Helix	3 α-Helix
**WT**	0.65	0.19	0.15	0.01	0.14	0.12	0.36	0.02
**E234P**	0.62	0.21	0.15	0.01	0.15	0.12	0.33	0.02
**R316Q**	0.62	0.23	0.14	0.02	0.13	0.12	0.34	0.03
**R322Q**	0.63	0.22	0.14	0.01	0.13	0.11	0.37	0.01
**C392D**	0.63	0.22	0.16	0.01	0.14	0.1	0.36	0.01

Table S6. Matrix values and cumulative percentage of WT and MT proteins

Variants	Covariance matrix	First 30 PCs	First 3 PCs
**WT**	68.2717	88.70	55.66
**E234P**	50.2536	87.85	54.69
**R316Q**	51.9791	86.59	50.35
**R322Q**	82.1308	105.45	61.32
**C392D**	64.1511	90.39	67.05

Table S7. Cosine content values of first 2 PCs

Variants		Cosine values
**WT**	PC1	0.03112
	PC2	0.00299
**E234P**	PC1	0.001338
	PC2	0.003291
**R316Q**	PC1	0.002928
	PC2	0.256055
**R322Q**	PC1	0.008445
	PC2	1.67E-07
**C392D**	PC1	0.039852
	PC2	0.08592

Table S8. Mean RMSDs and Rgs of N- and C-terminals

Variants	RMSD (nm)		Rg (nm)	
	N-Terminal	C-Terminal	N-Terminal	C-Terminal
**WT**	0.50	0.44	2.0	1.79
**E234P**	0.48	0.42	1.95	1.81
**R316Q**	0.46	0.39	1.96	1.80
**R322Q**	0.56	0.42	1.97	1.83
**C392D**	0.56	0.38	1.97	1.79

Table S9. Residues satisfying the condition $C_B^{WT} - C_B^{MT} \geq 0.02$

Variants	E234P	C392D
**Residue numbers**	69,72,98,236,241,284,288,294,296, 301,315,319,321,322,363,371,375, 378,380,381,384,391,403,434,437, 448,451,517	105,107,110,113,135,159,163,198, 200,202,204,229,231,234,241,243, 273,284,288,294,296,300,304,315, 319,321,324,363,383,391,399,406, 437,440,445,450, 454,467,513,517

Table S10. Molecular docking of WT- and MTs-6mA computed from 5 independent docking tools (Binding energies were measured in kcal/mol)

Variants	AutoDock Vina	QVina2	SMINA	LeDock	GNINA	Average
**WT**	-6.9	-6.1	-6	-7.2	-4.9	-6.2
**E234P**	-5.1	-4.9	-5.5	-5.4	4.2	-5.02
**R316Q**	-5.2	-5.3	-6	-5.1	-4	-5.1
**R322Q**	-6.2	-5.3	-5.8	-6.6	-4.4	-5.6
**C392D**	-5.8	-6	-5.3	-6.8	-3.8	-5.5
